# Pofut1 point-mutations that disrupt O-fucosyltransferase activity destabilize the protein and abolish Notch1 signaling during mouse somitogenesis

**DOI:** 10.1371/journal.pone.0187248

**Published:** 2017-11-02

**Authors:** Rieko Ajima, Emiko Suzuki, Yumiko Saga

**Affiliations:** 1 Mammalian Development Laboratory, Genetic Strains Research Center, National Institute of Genetics, Mishima, Shizuoka, Japan; 2 Mouse Research Supporting Unit, National Institute of Genetics, Mishima, Shizuoka, Japan; 3 Department of Genetics, SOKENDAI, Mishima, Shizuoka, Japan; 4 Gene Network Laboratory, Structural Biology Center, National Institute of Genetics, Mishima, Shizuoka, Japan; 5 Department of Biological Sciences, Graduate School of Science, The University of Tokyo, Bunkyo-ku, Tokyo, Japan; Medical College of Wisconsin, UNITED STATES

## Abstract

The segmental pattern of the vertebrate body is established via the periodic formation of somites from the presomitic mesoderm (PSM). This periodical process is controlled by the cyclic and synchronized activation of Notch signaling in the PSM. Protein O-fucosyltransferase1 (Pofut1), which transfers *O*-fucose to the EGF domains of the Notch1 receptor, is indispensable for Notch signaling activation. The *Drosophila* homologue Ofut1 was reported to control Notch localization via two different mechanisms, working as a chaperone for Notch or as a regulator of Notch endocytosis. However, these were found to be independent of *O*-fucosyltransferase activity because the phenotypes were rescued by Ofut1 mutants lacking *O*-fucosyltransferase activity. Pofut1 may also be involved in the Notch receptor localization in mice. However, the contribution of enzymatic activity of Pofut1 to the Notch receptor dynamics remains to be elucidated. In order to clarify the importance of the *O*-fucosyltransferase activity of Pofut1 for Notch signaling activation and the protein localization in the PSM, we established mice carrying point mutations at the 245^th^ a.a. or 370-372^th^ a.a., highly conserved amino-acid sequences whose mutations disrupt the *O*-fucosyltransferase activity of both *Drosophila* Ofut1 and mammalian Pofut1, with the CRISPR/Cas9 mediated genome-engineering technique. Both mutants displayed the same severely perturbed somite formation and Notch1 subcellular localization defects as the Pofut1 null mutants. In the mutants, Pofut1 protein, but not RNA, became undetectable by E9.5. Furthermore, both wild-type and mutant Pofut1 proteins were degraded through lysosome dependent machinery. Pofut1 protein loss in the point mutant embryos caused the same phenotypes as those observed in *Pofut1* null embryos.

## Introduction

The segmental feature of the vertebrate body is established in the embryo via the periodic formation of somites from the anterior unsegmented presomitic mesoderm (PSM). During somitogenesis, Notch signaling activation plays crucial roles to make the periodicity of somites and to define the segmental border in mice (Reviewed in [1, 2]). In the PSM, Notch signaling is activated via cleavage of Notch1 upon the binding of its ligand Dll1 expressed on a juxtaposed cell. After Notch1 cleavage at the cell membrane, the intracellular domain of Notch1 (NICD) translocates into nuclei to activate target genes, which in turn suppresses Notch signaling activation in trans via activation of *Lunatic fringe* (*Lfng*). Thus, the periodical somite formation is controlled by cyclic activation of Notch signaling and negative feedback by Notch targets in the PSM (Reviewed in [1, 2]). In order to activate Notch signaling in a group of cells periodically and synchronously in the PSM, cell surface amounts of Notch receptors and its ligands should be tightly regulated. It has been shown that subcellular localization of Notch receptors is controlled by several post-translational modifications, including proteolysis, ubiquitination, and glycosylation (Reviewed in [3–5]). Proteolysis of Notch receptors plays important roles for inducing endocytosis of receptors as well as ligands, which is essential for activation of the signaling [6, 7]. If the Notch receptors do not interact with ligands, receptors are endocytosed constitutively, and recycled or degraded in lysosomes. Ubiquitination of Notch receptors is also important for controlling the endocytosis of inactivated receptors and subsequent trafficking [8, 9]. The extracellular domain of Notch receptors is highly glycosylated during synthesis in the Endoplasmic Reticulum (ER) and trafficking through the Golgi. Glycosylation affects the maturation of Notch receptors, their trafficking, and their affinity for Notch ligands [10–12]. Previous observations demonstrated that Lfng plays a crucial role in synchronizing Notch signaling in the PSM [13, 14]. Lfng functions to elongate ß1,3 N-acetylglucosamine to *O*-fucose attached to EGF repeats of the Notch1 receptor [15, 16]. Taken together, glycosylation of Notch1 may be a critical modification for synchronous activation of Notch signaling.

The *O*-fucose attached to EGF repeats of the Notch1 receptor is transferred by Protein O-fucosyltransferase1 (Pofut1) [17, 18]. Pofut1 has been shown to be localized to the ER [19], and is indispensable for Notch signaling activation [17, 20, 21]. The importance of *O*-fucose modification for Notch1 signaling activation was demonstrated by the reduced ligand binding ability of Notch1 in *Pofut1* mutant ES cells without changing the cell surface amount of Notch1 protein [22]. Crystal structure analysis of Notch1 and its ligands also revealed that the *O*-fucose modification directly influences ligand binding [23, 24]. Furthermore, an *O*-fucosylation site mutation in the EGF repeat of Notch1, which is implicated in ligand binding, was found to create a hypomorphic allele as the compound mutants with Notch1 deletion mutants displayed similar phenotypes with Notch1 null mutants [25]. On the other hand, subcellular localization of Notch1 protein was greatly altered in the PSM of *Pofut1*-null embryos [26], suggesting that Pofut1 functions may be different in each cell/tissue, and that the control of ligand binding affinity by *O*-fucose modification of the Notch1 extracellular domain may not be the only function of Pofut1 in the PSM. Similar abnormal subcellular localization of Notch proteins was reported in *Ofut1*, the *Pofut1*-homologue, mutant in *Drosophila* [20, 27]. In *Drosophila*, there are different explanations for Ofut1functions in controlling Notch subcellular localization. One is that Ofut1 acts as a chaperone for Notch proteins in the ER [27], and the other is that it controls the endocytosis of Notch proteins [28]. In either case, each function is independent from the *O*-fucosyltransferase activity of Ofut1 as the function was rescued by expression of Ofut1^R245A^ [27] or the DXD-like motif mutant Ofut1^3G^ [28]. The Arg 245 of Ofut1/Pofut1 is conserved among species and has been shown as an important residue for enzymatic activity [22, 27, 29, 30]. The DXD-like motif is found not only in fucosyltransferases, but in many other glycosyltransferases in different species as well [31], and has been reported as important for enzymatic activity [20, 32].

It is possible that the important functions of Pofut1 as *O*-fucosyltransferase for periodical activation of Notch signaling in the PSM are masked by the phenotype that is induced by abnormal Notch1 localization in the *Pofut1*-null mutant. To investigate this possibility, we took advantage of the Clustered Regularly Interspaced Short Palindromic Repeats (CRISPR) /CRISPR-Associated Protein9 (Cas9) mediated genome-editing technique to establish Pofut1 point mutant mice that lack *O*-fucosyltransferase activity. This is the first report of mice lacking the enzymatic activity of Pofut1. By analyzing these mice, we unexpectedly found that the Pofut1 protein stability was markedly decreased in the mutants, and abnormal somitogenesis and mis-localization of Notch1 was observed, as in *Pofut1*-null mutants.

## Materials and methods

### Mice

Mice carrying *Pofut1*-null (*Pofut1*^Δ*/*Δ^) and *RBPJk*-null (*RBPJk* ^Δ/Δ^) alleles were established previously [26, 33]. B6C3F1 and MCH strain mice were purchased from CLEA Japan, Inc. Mice were kept in a specific-pathogen free (SPF) facility at 23 ± 2°C, under 50 ± 10% humidity and 12-hr (8 am-8 pm) lights on, 12-hr (8 pm-8 am) lights off conditions. Mice were kept in cages larger than 130 cm^2^ per mouse, and supplied CE-2 food (CREA Japan Inc) and water with 3 ppm sodium hypochlorite. The cages were changed every week. When mice were given surgery, they were anesthetized by intraperitoneal injection of 0.2 μg of 2,2,2-tribromoethyl alcohol (Avertin)/g body weight in advance. Mice were sacrificed with carbon dioxide gas or by cervical spine fracture dislocation. The monitoring of SPF conditions was conducted by the Central Institute for Experimental Animals with the core set every three months, and the full set once a year. All mouse experiments were approved by the Animal Experimentation Committee at the National Institute of Genetics (Permit Number: 27–13).

### Immunohistochemistry

Embryos were dissected in ice-cold PBS, fixed with 4% PFA/PBS for 1 hr on ice, submerged in 10%, 20%, and 30% Sucrose/PBS at 4°C, then embedded in O.C.T. compound (Sakura Finetek) and frozen. The frozen sections (7-μm thickness) were blocked with 3% BSA/PBS at room temperature for 1 hr and incubated overnight at 4°C in a mixture of the following primary antibodies with indicated dilutions in 3% BSA/PBS: anti-Notch1 (C20-R, 1:500, Santa Cruz Biotechnology) and anti-Pan-Cadherin (CH-19, 1:500, Sigma), or anti-KDEL (10C3, 1:500, Stressgen). After washing the sections with 0.1% Tween-20/PBS three times, the sections were incubated with Alexa Fluor 488-conjugated anti-rabbit IgG (H+L) antibody (1:800, Life Technologies), Alexa Fluor 594-conjugated anti-mouse IgG (H+L) antibody (1:800, Life Technologies), and Hoechst 33342 (1:1000, Calbiochem) in 3% BSA/PBS for 2 hrs at room temperature. After extensive washing with 0.1% Tween-20/PBS, the sections were mounted. NICD was stained as previously reported [13]. These sections were observed using FluoView FV1200 laser scanning confocal microscopy with the IX83 (Olympus). More than three embryos were used for each experiment unless otherwise specifically stated.

### Transmission electron microscopy (TEM) and Immuno-TEM

TEM was conducted according to the protocol previously published [34] with some modifications. Briefly, embryos for TEM analysis were dissected in ice-cold PBS, then immediately fixed with 2% formaldehyde and 2.5% glutaraldehyde in 0.1 M phosphate buffer (PB) for 2 hrs at room temperature, washed with 3% Sucrose /0.1M PB, post-fixed with 1% OsO_4_/0.1M PB, washed with distilled water, block-stained with 0.5% aqueous uranyl acetate, dehydrated with a series of ethanol, then embedded in Epon-mixture. The samples were trimmed with a glass knife, then cut to make ultra-thin sections with a Leica EM UC6. The sections were collected on grids, and stained with 2% uranyl acetate and Reynolds’ lead solution. Immuno-TEM was conducted according to the protocol previously published [35] with some modifications. Briefly, embryos for Immuno-TEM analysis were dissected in ice-cold PBS, then immediately fixed with periodate-lysine-paraformaldehyde fixative for 2 hrs at room temperature, submerged in 10%, 20%, and 30% Sucrose/PBS at 4°C, then embedded in O.C.T. compound (Sakura Finetek) and frozen. The frozen sections (10-μm thickness) were treated with 150 mM glycine/PBS, blocked with 3% BSA/PBS at room temperature for 1 hr, and incubated with anti-Notch1 (C20-R, 1:100, Santa Cruz Biotechnology) antibody three days at 4°C. After washing the sections with PBS five times, they were incubated with goat anti-rabbit IgG HRP-conjugated antibody (1:200, Cell Signaling) for 2 hrs at room temperature. After washing with PBS, the sections were re-fixed with 0.5% glutaraldehyde/PBS for 5 minutes, washed with PBS, and incubated with 0.025% 3,3’-diaminobenzidine 4HCl (Dojindo) in 50 mM Tris-HCl pH 7.6 (DAB solution) for 30 min, then incubated with DAB solution containing 0.005% H_2_O_2_. The DAB reaction was stopped by washing the sections with PBS several times. Then, the sections were treated with 2% OsO4/0.1M PB for 1 hr, dehydrated with an ethanol series, and embedded with Epon-mixture. The samples were cut to make ultra-thin sections with a Leica EM UC6. The sections were observed, and pictures were taken with low (x10000) and high (x30000) magnification using the JOEL JEM 1010. The quantification of the subcellular localization was conducted as follows. Cells with at least partially visible nuclei in the low-magnification TEM images were counted, and subcellular localization was confirmed using high-magnification images of the same area. The localization was categorized as on the cell surface, on the rough-ER, or on vesicles not in the rough-ER. The cells containing vesicles not in the rough-ER without cell surface and rough-ER signal markers were counted as “other vesicles only”. The ratios of each category were calculated for each embryo.

### Preparation of sgRNA and hCas9 expression vector, sgRNA, mRNA, and ssODN

The bicistronic expression vector expressing sgRNA and hCas9 mRNA (pX330) [36] was purchased from Addgene (Cambridge, MA). pX330 was linearized with BbsI, gel purified, then ligated with an annealed pair of oligos for R245A (5’- caccGTGGGCATTCATCTGCGCAT-3’ and 5’- aaacATGCGCAGATGAATGCCCAC-3’) and 3G (5’- caccGTGCCTTCGTGAAGCGGGAGC-3’ and 5’- aaacGCTCCCGCTTCACGAAGGCAC-3’), and the resulting vectors were designated as pX330/R245A and pX330/3G, respectively. pT7-sgRNA [37] was linearized with BbsI, gel purified, then ligated with an annealed pair of oligos for R245A (5’- agggGTGGGCATTCATCTGCGCAT-3’ and 5’- aaacATGCGCAGATGAATGCCCAC-3’), and the resulting vector was designated as pT7/R245A. pT7/R245A and pX330/3G were then used as a template to amplify the PCR product with the forward primer for R245A (5’- TAATACGACTCACTATAGGGGTGGG-3’or T7 promoter-attached forward primer for 3G (5’- TTAATACGACTCACTATAGGGGTGCCTTCGTGAAGCGGGAGC-3’), respectively, and common reverse primer (5’-AAAAAAGCACCGACTCGGTG-3’). The T7-sgRNA PCR products were gel purified and used as a template for in vitro transcription using MEGAshortscript T7 kit (Life Technologies). hCas9 mRNA was synthesized using the mMESSAGE mMACHINE SP6 Transcription Kit (Life Technologies) with linearized pSP64-hCas9 [38] as a template. The synthesized sgRNAs and mRNA were purified by phenol-chloroform-isoamylalcohol extraction, chloroform extraction, and isopropanol precipitation. The ssODNs were ordered from Gene Design, Inc. or Integrated DNA Technologies.

### One-cell embryo injection

B6C3F1 (C57BL/6N X C3H/HeN) female mice were superovulated and mated with B6C3F1 males, and fertilized embryos were collected from oviducts.

Different concentrations of vectors, sgRNA, hCas9 mRNA, and oligos were mixed in injection buffer (10 mM Tris-HCl and 0.1 mM EDTA, pH 7.5) and injected into the pronucleus of fertilized eggs in M2 medium (Sigma). The injected zygotes were cultured in KSOM (Millipore) at 37°C under 5% CO2 until the 2-cell stage after 1.5 days. Thereafter, 20–32 2-cell stage embryos were transferred into the uterus of pseudo-pregnant MCH females at 0.5 dpc. Embryos were dissected 9 days after transfer or born naturally. Two independent mouse lines of *Pofut1*^*R245A*^ or *Pofut1*^*3G*^ were established, and these mice were back-crossed with C57BL6/J more than 4 times to reduce off target mutation effects, and no change in *Pofut1*^*R245A/R245A*^ or *Pofut1*^*3G/3G*^ embryo phenotypes was confirmed after back-crossing.

### Genotyping by RFLP analysis and direct sequencing

For RFLP analysis, the PCR product of the *Pofut1*^*R245A*^ allele with R245A Fw (5’- ATGGTGCAATGCCATCCTTTAT-3’) and R245A Rv (5’- CATGTGCAAACACAAGGGTACAA-3’) was digested by MscI and run on a 1.5% Agarose gel with 1xTAE. The PCR product of the *Pofut1*^*3G*^ allele with 3G Fw (5’- CCAATCTTCATCCTCTCCAACCT-3’) and 3G Rv (5’- GGCTGCTTTGTATGTGCATGAT-3’) was digested by StuI and run on a 1.5% Agarose gel with 1xTAE. For direct sequencing, approximately 100 ng of the PCR products, the same products for RFLP analysis, was treated with ExoSAP-IT (Affymetrix, Inc.), reacted with the Big Dye Terminator v3.1 Cycle sequencing kit (Applied Biosystems) with R245A Seq. Fw (5’- TATCCCGTGGGAGTGGCAGC-3’), R245A Seq. Rv (5’- CAAATGGCAGCTGTTAAATG-3’), 3G Seq. Fw (5’- ctcttggtctgaaacttcag-3’), or 3G Rv primers, according to the manufacturer’s instructions, then analyzed with the ABI 3130xl genetic analyzer (Applied Biosystems).

### Mouse monoclonal antibody production

Synthetic peptides corresponding to amino acids 381–393 in mouse Pofut1 (CFGMDRPSQLRDEF), and amino acids 29–35 and 387–393 in mouse Pofut1 connected with Cysteine (RSAGSWDCSQLRDEF), were used as the antigens. The monoclonal antibodies were produced by Dr. Kurihara (Yokohama National University) and the detailed method was described previously [39]. We obtained clone #10–18 with the former peptide, and clones #28–11, #28–33, and #35–8 with the latter peptide.

### Western blot analysis

Whole embryos, ES cells, or 293T cells transfected with expression vectors using Lipofectamine 2000 (Invitrogen) were washed with PBS, and lysed with RIPA buffer (50 mM Hepes pH 7.4, 150 mM NaCl, 1% TritonX-100, 0.02% Sodium deoxycolate, and 1 mM EDTA) supplemented with Complete mini (Roche). The protein concentration was calculated with a Protein Assay (Bio-Rad) and samples were adjusted to be the same concentration. Sample buffer was added to the lysates and boiled for 5 min. Proteins were separated by running on an 8% SDS-PAGE gel, and transferred to an Immobilon-P membrane (Millipore). The membranes were blocked with 3% skim milk in TBS-T for 2 hrs at room temperature, and incubated with the following primary antibodies: anti-Notch1 (C20-R, 1:1000, Santa Cruz Biotechnology), anti-ß-catenin (rabbit polyclonal, 1:3000, Upstate Biotechnology), anti-Pofut1 ascites described above (1:2000), anti-ß-tubulin (TUB2.1, 1:5000, Sigma Aldrich), and HRP-conjugated anti-Flag antibody (Flag M2, 1:3000, Sigma Aldrich). For detecting endogenous Pofut1 protein, both #28–33 and #35–8 ascites were used for each experiment. The membranes were washed and incubated with HRP-conjugated anti-rabbit IgG antibody (1:3000, Cell Signaling) or HRP-conjugated anti-mouse IgG antibody (1:3000, Cell Signaling). The membranes were washed and incubated with SuperSignal West Femto Maximum Sensitivity Substrate (Thermo Scientific) and the signals were detected with an ATTO Ez-capture MG image analyzer. Signal intensities of Pofut1 and ß-tubulin bands were measured with ImageJ, background signals subtracted, and Pofut1 intensity was divided by the corresponding ß-tubulin intensity. Then, each signal was adjusted with the wild-type sample (in different dose experiments: vehicle treated sample; in time course experiments: 0-hr time point) as 1.

### Semi-quantitative real-time RT-PCR

Total RNA was isolated from whole embryos at E8.5 and E9.5 stages using an RNeasy mini kit (QIAGEN) according to the manufacturers' instructions. For the reverse transcription reaction, 0.5 μg of total RNA was used with SuperscriptIII (Invitrogen) and oligo-dT primers. To analyze the expression of *Pofut1* and *Hprt*, the following primer sets were used, respectively. *Pofut1* exon6 F (5’- TGAGAGCTACGTGTCAGAGATCCA-3’) and exon7 R (5’- CACAGTTTCCAATGAAGTGGTCAG-3’). *Hprt* F (5’- TCCTCCTCAGACCGCTTTT -3’) and R (5’- CCTGGTTCATCATCGCTAATC -3’). The house keeping gene *Hprt* was used as the “reference” gene. PCR reactions were carried out in 96-well microtiter plates in a 20-μl reaction volume with KAPA SYBRs FAST Universal 2X qPCR Master Mix (KAPABIOSYSTEMS). A Thermal Cycler Dice Real Time System (TAKARA) was programmed for an initial step of 96°C for 30 sec, followed by 40 cycles of 96°C for 5 sec, and 60°C for 30 sec. The relative expression level was calculated by the ΔΔCt method, and normalized with reference gene expression. Specificity of PCR amplification of each primer pair was checked in advance by running the PCR product on an agarose gel, and confirmed by melting curve analysis.

### Vector construction

To establish the Pofut1 expression vector, total RNA was obtained from whole embryos of C57BL6J at E11.0, reverse transcribed as described above, and the first strand DNA was used as a template. The *Pofut1* cDNA fragment was amplified and cloned into the pcDNA3.1(+) vector (Invitrogen). The Flag-tagged Pofut1 expression vector was established by inserting the 3xFlag sequence downstream of the putative signal peptide sequence, 89 bp downstream of the first Met. To establish point mutant Flag-tagged Pofut1 expression vectors, mutated expression vectors were amplified by PCR using the Flag-tagged Pofut1 expression vector as a template with the following primer sets. For Pofut1 aaR245A: Pofut1 R245A F(5’-GCCattggctccgactggaa-3’) and Pofut1 aa244 R (5’-cagatgaatgcccacatagg-3’). For Pofut1 3G: Pofut1 aa370(3G) F (5’-GGGGGGGGcctccatgggag-3’) and Pofut1 aa369 R (5’-Ccgcttcacgaaggcagtga-3’). The resulting PCR products were ligated with DNA ligation kit ver.2 (TaKaRa). The PiggyBac vectors, pBase, pPBCAG-rtTAM2-IN and pPB-hCMV-1cHApA, were obtained from Dr. Hitoshi Niwa (Kumamoto Univ). A Flag-tagged activated mouse Notch1 cDNA fragment was amplified by PCR using the pME-FNIC vector, which was obtained from Dr. Ryoichiro Kageyama (Kyoto Univ.), as a template with the following primer sets: EcoRI-Flag activated-Notch1 F (5’-TTTTGGCAAAGAATTCACCATGGACTACAAAGACGAT-3’) and NotI-Notch1 R (5’- GCTTATCGAGCGGCCGCTTATTTAAATGCCTCTGGAATGTGGGTGAT-3’). The resulting PCR product was digested with EcoRI and NotI, and cloned into the EcoRI-NotI site of the *pPB-hCMV-1cHApA* vector, and designated as pPB-hCMV-active Notch1pA.

### Establishment and culture of ES cell lines

E3.5 stage embryos were obtained from natural mating of heterozygous mice of each Pofut1 mutant, flushed from the uterus, and cultured on feeder cells (primary mouse embryonic fibroblasts treated with Mitomycin C) in 48-well plates with 2i+LIF medium (ESGRO Complete Basal Medium (Millipore) supplemented with 3 μM CHIR99021 (Wako), 0.4 μM PD0325901 (Wako), 1000 U/ml LIF (Wako), and 1x Penicillin-Streptomycin (Gibco). Four to five days later, the hatched inner cell mass was trypsinized, and seeded on feeder cells with 2i+Lif medium. Once the cells were expanded to 12-well plates, the culture medium was switched to ES cell medium (DMEM high glucose (Gibco) supplemented with 20% FBS, 2 mM L-glutamine (Gibco), 1x MEM NEAA (Gibco), 1x Penicillin-Streptomycin (Gibco), 55 μM 2-Mercaptoethanol (Gibco), 1000 U/ml LIF (Wako), 3 mM Adenosine; Cytidine; Guanosine; Uradine, and 1 mM Thymidine (Sigma)). Activated Notch1 inducible ES cell lines were established by transfection of pBase, pPBCAG-rtTAM2-IN, and pPB-hCMV-active Notch1pA vectors with Lipofectamine 2000 (Invitrogen), and selected with 150 μg/ml G418 (Gibco) for 1 week, and surviving colonies combined. When each ES cell line was used for experiments, the feeder cells were depleted by seeding on a 0.1% gelatin coat cell culture dish for 1 hr at 37°C, then ES cells were further expanded on a 0.1% gelatin coat cell culture dish. MG132 (PEPTIDE) and Chloroquine (Sigma) were dissolved in DMSO (Sigma).

### Immunocytochemistry

ES cells were seeded on feeder cells in a Chamber slideII (IWAKI), treated with/without 5 μM Chloroquine (Sigma) and 1 μM Lysotracker Red-DND99 (Invitrogen) for 8 hrs, fixed with 4% PFA/PBS, permeabilized with 0.2% TritonX-100/PBS, and then blocked with 3% BSA/PBS. Antibody incubation and detection were the same as the immunohistochemistry protocol described above.

### Whole embryo culture

E9.5 stage MCH embryos were harvested in M2 medium (Sigma) supplemented with 10% FBS, and rotation cultured in 75% rat serum, 25% DMEM (Invitrogen) with or without DAPT (Selleckchem) for 8 hrs.

## Results

### Establishment of Poftu1 mutant mice deficient for O-fucosyltransferase activity via CRISPR/Cas9 mediated genome editing

**Fig 1:**
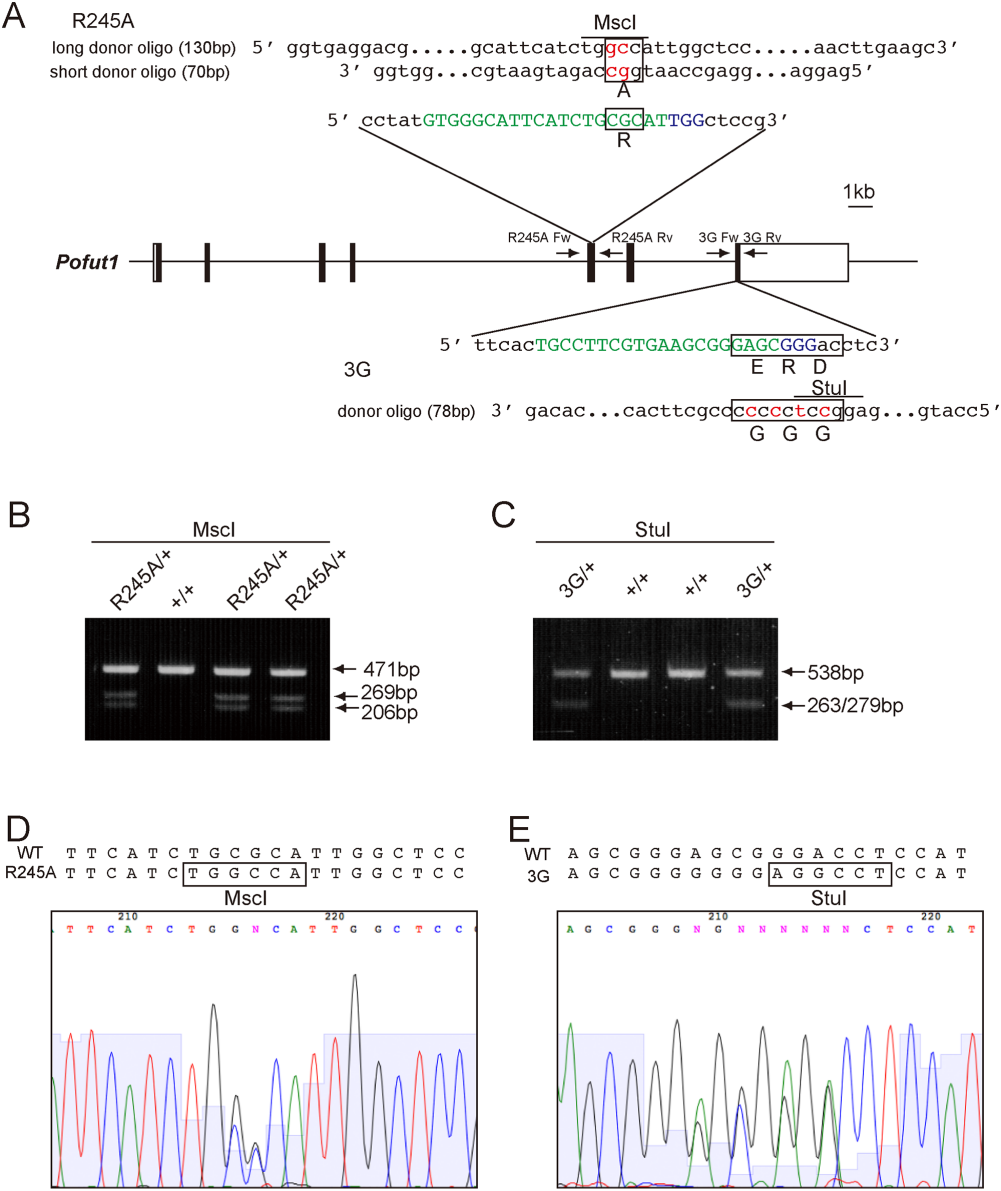
Strategy for introducing a point mutation in the *Pofut1* locus using the CRISPR/Cas9 mediated genome-editing technique. (A) Schematic of the two independent point mutation sites of the *Pofut1* gene. The first mutation was introduced in the 5^th^ exon, and the resulting allele carried the R245A mutation. The second mutation was introduced in the 7^th^ exon and the resulting allele carried the ERD into GGG (3G) mutation. Each sgRNA coding sequence is capitalized and labeled in green. The protospacer-adjacent motif (PAM) sequence is capitalized and labeled in blue. Two donor oligos and one donor oligo were used for the R245A site and 3G site, respectively. The introduced mutation is indicated in red in the donor oligo sequence, and resulting mutant amino acids are shown below the boxes. Restriction enzyme sites, MscI and StuI, which were created by the mutations, are shown with lines above the donor oligo sequence. PCR primers (R245A Fw, R245A Rv, 3G Fw, and 3G Rv) used for PCR genotyping are shown as arrows. (B) RFLP analysis of F1 embryo samples. Amplified PCR product using primers R245A Fw and R245A Rv, followed by digestion with MscI. The genotypes shown above were genotyping results by direct sequencing. (C) RFLP analysis of F1 embryo samples. Amplified PCR product using primers 3G Fw and 3G Rv, followed by digestion with StuI. The genotypes shown above were genotyping results by direct sequencing. (D,E) Direct sequencing results of PCR products using primers R245A Fw and R245A Rv, and primers 3G Fw and 3G Rv, respectively.

In order to assess the importance of the *O*-fucosyltransferase activity of Pofut1, we established point mutation mice by CRISPR/Cas9 mediated genome editing. Two independent sites, Arg 245 and the DXD-like motif ERD, were chosen as target sites. The sgRNA for each target was selected using the “CRISPR design” tool (http://crispr.mit.edu) [40] (Fig 1A). To generate the 245^th^ arginine into alanine (R245A)-point mutant mice, we compared two approaches; one was injecting in vitro transcribed R245A sgRNA and *hCas9* mRNA with single-strand oligo deoxynucleotides (ssODN) as a donor oligo [41], and the other was injecting the circular bicistronic expression vector pX330/R245A, and expressing R245A sgRNA and *hCas9* mRNA with ssODN [37]. We also compared the targeting efficiency with long and short donor oligos, which had 64 bp, and 34 bp homology arms, respectively, with a 2 bp mutation site in the middle of the ssODN (Fig 1A). As *Pofut1*-null embryos exhibit embryonic lethality around E10, we conducted the F0 generation analysis by dissecting mutant embryos at E9.5. The efficiency of gene modification, including non-homologous end joining (NHEJ) mediated non-specific insertion and deletion (in-del) mutations, and homology-directed repair (HDR) mediated knock-in, was higher in the case of injecting transcribed RNA (9 out of 11 embryos) than injecting pX330/R245A (8 out of 17 embryos by injecting in 5 ng/μl, and 2 out of 8 embryos by injecting in 2 ng/μl) (Table 1). Injecting pX330/R245A induced more mosaicism than injecting transcribed RNA. Knock-in of the point mutation occurred even using short donor ssODN (7 out of 18 embryos or pups) with comparable frequency to using long donor ssODN (3 out of 6 embryos) (Tables 1 and 2). We obtained 9 homozygous mutant embryos, including 7 embryos that had the correctly knocked-in point mutation in both alleles (3 embryos) or one of two alleles (4 embryos). Most of the homozygous mutant embryos (8 out of 9 embryos) phenocopied *Pofut1*^Δ*/*Δ^ embryos with differing ranges of severity, suggesting that *Pofut1*^*R245A/R245A*^ may display embryonic lethality. Indeed, we never obtained homozygous mutant pups when we tried to establish a *Pofut1*^*R245A*^ mutant line (Table 2). Two *Pofut1*^*R245A*^ mutant pups were obtained by injecting the lower (2 ng/μl) concentration of pX330/R245A (Panels A-H in S1 Fig, Table 2). Some pups displayed several common phenotypes such as a small body, kinked tail, and coarse hair (Panels C and D in S1 Fig). Direct sequencing analysis of these pups revealed that the mosaicism, which may have been caused by introducing the mutation later than the two-cell stage. The pups with lower wild-type allele contribution, which were likely to contain homozygously mutated cells, corresponded with the severity of the phenotypes (Panels G and H in S1 Fig).

**Table 1:** F0 analysis of *Pofut1* gene targeted embryos.

Dose of RNA or vector	Donor length (100 ng/μl)	Injected	Two-cell	Gene modified/ embryos	Hetero:Homo	Knock-in/ Gene modified
50 ng/μl R245A sgRNA 100 ng/μl hCas9 mRNA	Short	108	56	9/11 (81.8%)	4:5	4/9 (44.4%)
5 ng/μl pX330/R245A	Short	31	12	3/7 (42.6%)	2:1	1/3 (33.3%)
2 ng/μl pX330/R245A	Short	34	10	1/4 (25.0%)	1:0	0/1 (0%)
5 ng/μl pX330/R245A	Long	128	53	5/10 (50.0%)	2:3	3/5 (60.0%)
2 ng/μl pX330/R245A	Long	62	21	1/4 (25.0%)	1:0	0/1 (0%)
total		363	152	19/36 (52.8%)	10:9	8/19 (42.1%)

Indicated RNA or vector and ssODN mixture was injected into zygotes, two-cell embryos were transferred into pseudo-pregnant females, and embryos were dissected 9 days after transfer at stage E9.5. Genotype of all embryos was analyzed by direct sequencing, and embryos that had the wild-type allele are indicated as Hetero.

**Table 2:** Generation of *Pofut1*^*R245A*^ mutant mice.

Dose of RNA or vector	Donor length (100 ng/μl)	Injected	Two-cell	Gene modified/ pups	Hetero:Homo	Knock-in/ Gene modified
50 ng/μl R245A sgRNA 100 ng/μl hCas9 mRNA	Short	59	30	0/3 (0%)	-	-
2 ng/μl pX330/R245A	Short	102	62	5/9 (55.6%)	5:0	2/5 (40.0%)
5 ng/μl pX330/R245A	Long	336	152	0/6 (0%)	-	-
2 ng/μl pX330/R245A	Long	50	33	0/5 (0%)	-	-

Indicated RNA or vector and ssODN mixture was injected into zygotes, and two-cell embryos were transferred into pseudo-pregnant females. Genotype of all pups was analyzed by direct sequencing.

Next, we established *Pofut1*^*3G*^ mutant lines in which the DXD-like motif, tripeptide ERD, was mutated into GGG (3G) by injecting transcribed 3G sgRNA and *hCas9* mRNA with donor ssODN (Fig 1A). We could not obtain homozygous mutant mice by F0 generation analysis at the E9.5 stage, presumably because of the lower activity of double-strand breaking by the sgRNA and Cas9 for this locus. Instead, we obtained 4 heterozygous knock-in pups (Panels J and K in S1 Fig, Table 3). It has been reported that off-target mutations may occur using the CRISPR/Cas9 system. Previous studies demonstrated that loci with mismatches between sgRNA and target DNA sequences in the “seed sequence”, which is 8–12 bp upstream of the PAM sequence, were tolerated for the double strand break by the CRISPR/Cas9 system [36, 40, 42, 43]. We analyzed potential off-target sites that did not contain mismatches 8 bp upstream of the PAM with an NGG sequence or less than two mismatches in the entire 3G sgRNA sequence for mutations in the F0 generation *Pofut1*^*3G*^ pups by direct sequencing. However, we could not find any mutations (primer pairs used for this analysis are shown in S1 Table). No off-target sites that fall into the categories shown above were observed for R245A sgRNA.

**Table 3:** Generation of *Pofut1*^*3G*^ mutant mice.

Dose of RNA and ssODN	Stage	Injected	Two-cell	Gene modified/ organism	Hetero:Homo	Knock-in/ Gene modified
50 ng/μl 3G sgRNA 100 ng/μl hCas9 mRNA 100 ng/μl Donor ssODN	E9.5	248	112	7/10 (70.0%)	7:0	4/7 (57.1%)
	pups	325	172	8/18 (44.4%)	8:0	4/8 (50.0%)

Indicated RNA and ssODN mixture was injected into zygotes, and two-cell embryos were transferred into pseudo-pregnant females. Genotype of all embryos and pups was analyzed by direct sequencing.

### *Pofut1*^*R245A/R245A*^ and *Pofut1*^*3G/3G*^ embryos phenocopied Pofut1 null embryos

**Fig 2:**
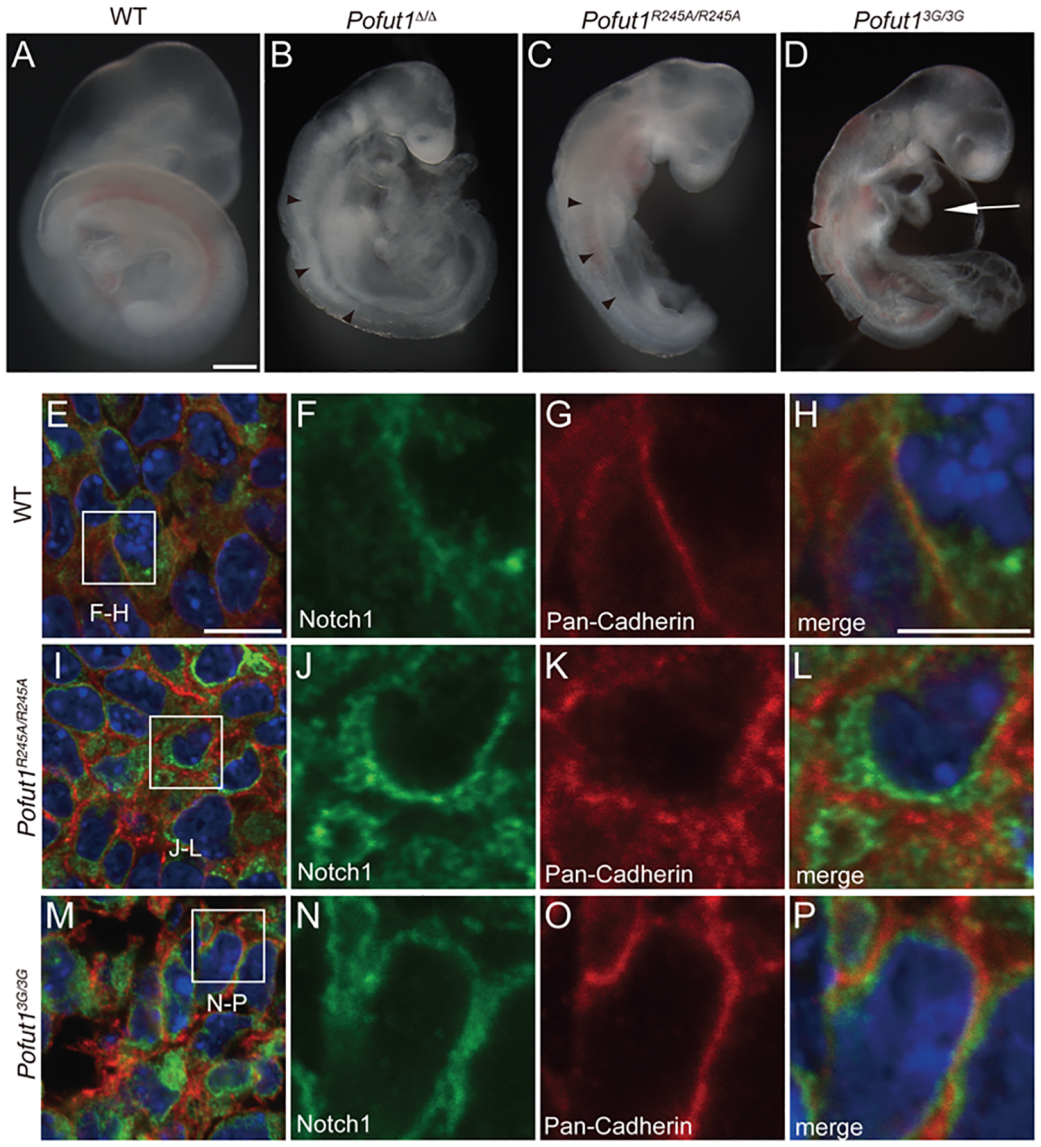
*O*-fucosyltransferase deficient Pofut1 mutant embryos phenocopied Pofut1 null mice. (A-D) WT, *Pofut1*^Δ*/*Δ^, *Pofut1*^*R245A/R245A*^, and *Pofut1*^*3G/3G*^ embryos at stage E9.5 are shown. Abnormal somites are shown with arrowheads, and an abnormally enlarged cardiac cavity is shown with an arrow. Scale bar = 500μm (A) (E-P) Immunohistochemistry of anterior PSM in E9.5 WT (E-H), *Pofut1*^*R245A/R245A*^ (I-L), and *Pofut1*^*3G/3G*^ (M-P) embryos using anti-Notch1 (green), anti-Pan-cadherin (red: cell surface) antibodies, and Hoechst33324 (blue: Nuclei). The three right panels are magnified images of insets shown in E, I, and M. Scale bars = 10 μm (E), and 5 μm (H).

*Pofut1*^*R245A*^ and *Pofut1*^*3G*^ mutants were backcrossed with wild-type C57BL6/J mice, and pups were genotyped by RFLP (Fig 1B and 1C) and/or direct sequencing (Fig 1D and 1E). *Pofut1*^*R245A*/+^ and *Pofut1*^*3G/+*^ mice were normal and fertile. These heterozygous mice were intercrossed to collect embryos. Both *Pofut1*^*R245A*/*R245A*^ and *Pofut1*^*3G/3G*^ embryos displayed the same phenotypes reported in *Pofut1* null mutants [21], such as small and disorganized somites, small head, and abnormal heart looping, sometimes exhibiting an enlarged cardiac cavity (Fig 2A–2D), indicating that the mutation compromised Pofut1 function.

Next, to assess the impact of the mutation we introduced, we examined Notch1 localization using immunohistochemistry. In wild-type embryos, Notch1 protein was localized on the cell surface (Fig 2E–2H, Panels A-D in S2 Fig) as well as intracellularly (Panels I-L in S2 Fig) as small dots in the PSM of mouse embryos. In Pofut1 null (*Pofut1*^Δ*/*Δ^) embryos, Notch1 protein no longer localized on the cell surface (Panels E-H in S2 Fig) and accumulated intracellularly (Panels M-P in S2 Fig), as reported before [26]. In *Pofut1*^*R245A*/*R245A*^ and *Pofut1*^*3G/3G*^ embryos, Notch1 protein also accumulated intracellularly in the PSM (Fig 2I–2L and 2M–2P), as observed in *Pofut1*^Δ*/*Δ^ embryos (Panels E-H in S2 Fig). Most of the Notch1 protein in *Pofut1*^Δ*/*Δ^, *Pofut1*^*R245A*/*R245A*^, and *Pofut1*^*3G/3G*^ embryos was not matured by the first proteolytic cleavage step (S-1 cleavage), which normally occurs in the Golgi apparatus, suggesting that Notch1 protein accumulated in the ER (Panel A in S3 Fig).

**Fig 3:**
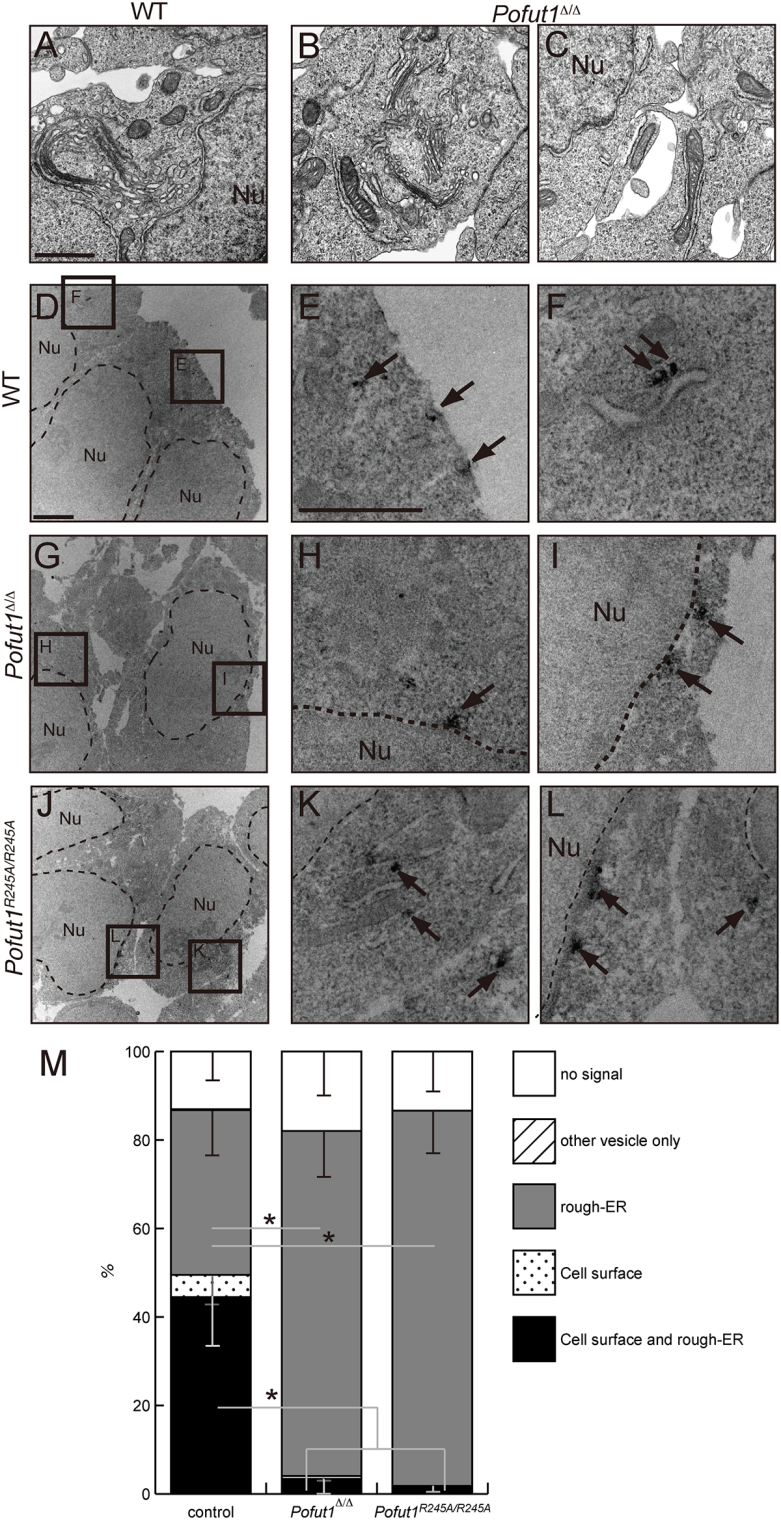
Immuno-TEM analysis revealed Notch1 protein accumulation in the ER in *Pofut1* mutant PSM. (A-C) TEM analysis of anterior PSM in E8.5 wild-type (A) and *Pofut1*^Δ*/*Δ^ (B and C) embryos. Scale bars = 1 μm (A). (D-L) Immuno-TEM analysis of anterior PSM in E8.5 wild-type (D-F), *Pofut1*^Δ*/*Δ^ (G-I), and *Pofut1*^*R245A/R245A*^ (J-K) embryos using anti-Notch1 antibody. Scale bars are 2 μm (D) and 1 μm (E). The boxes in D, G, and J indicate the positions of enlarged images in E, F, H, I, K, and L. Arrows indicate Notch1 signals. Nuclei are indicated as “Nu”. (M) Quantification of subcellular localization of Notch1 in anterior PSM of control (5 embryos, n = 23, 33, 35, 38, and 82 cells), *Pofut1*^Δ*/*Δ^ (3 embryos, n = 46, 37, 51 cells), and *Pofut1*^*R245A/R245A*^ (3 embryos, n = 34, 55, 55 cells). Average ratios of each subcellular localization are shown as a bar graph. Control contained wild-type and heterozygous samples of each genotype. Asterisks indicate P<0.01; paired t-test.

Even though the majority of Notch1 protein was reported to accumulate in the endoplasmic reticulum (ER) in the PSM of *Pofut1*^Δ*/*Δ^ embryos [26], Notch1 protein was not completely merged with the ER marker (Panels M-P in S2 Fig). To assess the localization of Notch1 more precisely, we conducted analyses with transmission electron microscopy (TEM). Gross appearance of each organelle, including the ER, was normal in the PSM in both WT and *Pofut1*^Δ*/*Δ^ embryos (Fig 3A–3C, Panels A and B in S4 Fig), except for slight swelling in some cristae of mitochondria only observed in *Pofut1*^Δ*/*Δ^ embryos at the E9.5 stage (Panel D in S4 Fig). Immuno-TEM analysis confirmed Notch1 localization on the cell surface (Fig 3E and 3M: 49.5±17.5%), and intracellularly such as on the vesicles (Fig 3E) and rough-ER (Fig 3F and 3M: 81.7±21.2%). In the PSM of *Pofut1*^Δ*/*Δ^ embryos, Notch1 was mainly localized on the perinuclear ER (Fig 3G–3I and 3M: 81.4±13.5%), and rarely observed on the cell surface (Fig 3M: 4.0±4.2%). Notch1 in *Pofut1*^*R245A*/*R245A*^ embryos was also observed in the ER similar with *Pofut1*^Δ*/*Δ^ embryos (Fig 3J–3L and 3M: 86.6±11.8%). These results indicate that Notch1 is accumulated in the perinuclear ER in *Pofut1*-null and *Pofut1*^*R245A*/*R245A*^ embryos.

### Pofut1^R245A^ and Pofut1^3G^ proteins were down-regulated by post-transcriptional regulation

**Fig 4:**
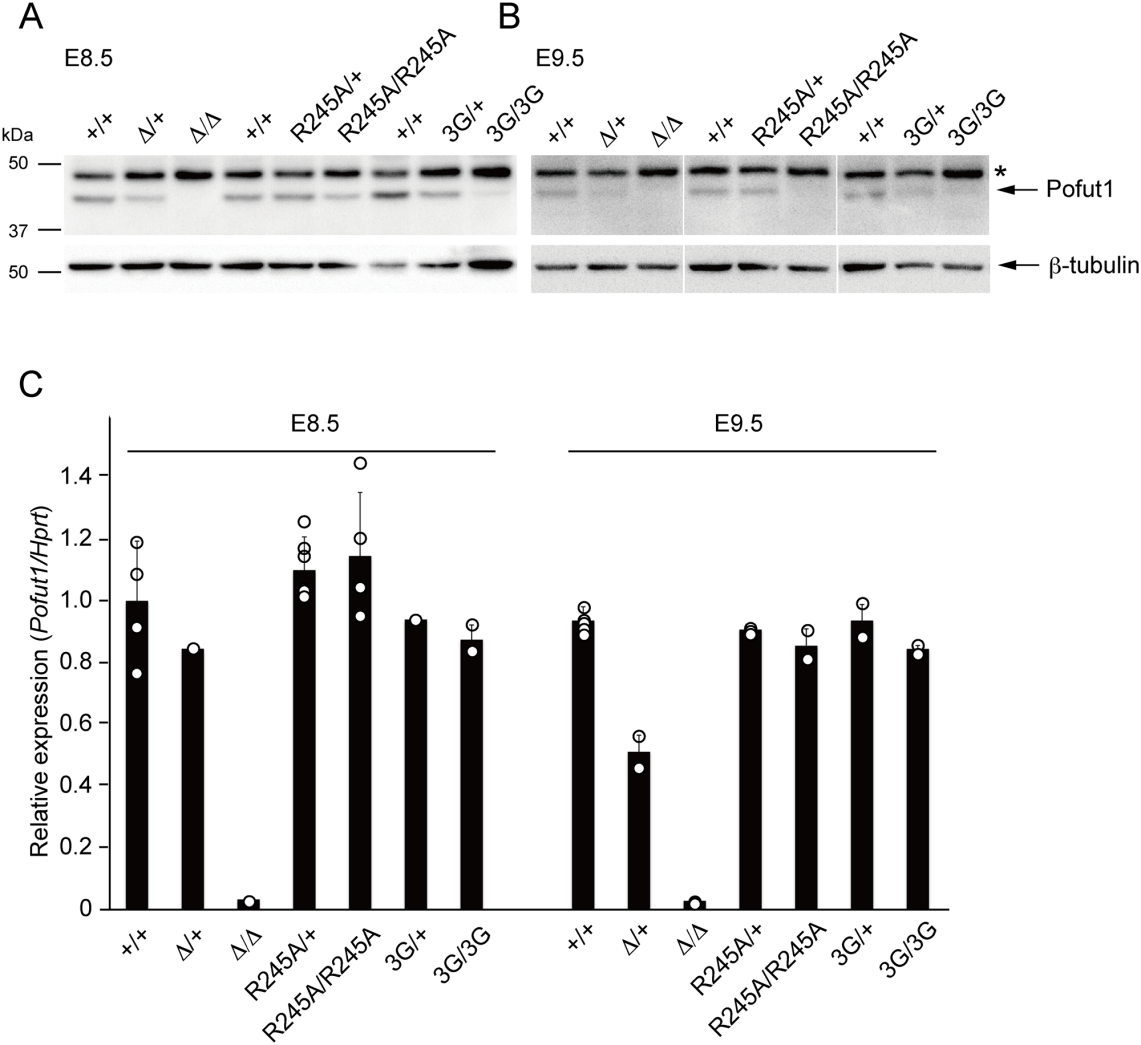
Pofut1 protein, but not RNA, was reduced in O-fucosyltransferase-deficient Pofut1 mutant embryos. (A and B) Upper panels show western blot analysis of Pofut1 expression using whole embryos of indicated genotypes at E8.5 (A) and E9.5 (B). Lower panels show the ß-tubulin amount as a loading control. (C) Semi-quantified RT-PCR results using whole embryos of indicated genotypes at E8.5 and E9.5 are shown. *Pofut1* mRNA amount was normalized with the *Hprt* mRNA amount. The RNA amount in individual embryos was shown as circles and the average expression levels were shown as a bar graph.

In order to examine the expression level of Pofut1 protein, we produced mouse monoclonal antibodies against Pofut1 peptides. After screening the clones, ascites from each clone were used for western blot analysis using whole lysate of *Pofut1*^*+/+*^, *Pofut1*^Δ*/+*^, and *Pofut1*^Δ*/*Δ^ embryos. We obtained 4 clones that can detect endogenous Pofut1 protein by western blot (Panels A-D in S5 Fig). Immunoreactivity of these antibodies for wild-type, and Pofut1^R245A^ and Pofut1^3G^ mutant proteins were examined using cell lysates that expressed Flag-tagged Pofut1 protein, and confirmed equivalent reactivity of these antibodies for wild-type and mutant Pofut1 proteins (Panels E-I in S5 Fig). These antibodies were used to examine Pofut1 protein expression in each mutant, and both Pofut1^R245A^ and Pofut1^3G^ protein levels were decreased in *Pofut1*^*R245A/+*^ *and Pofut1*^*3G/+*^ embryos, respectively, yielding approximately half the amount of Pofut1 protein than in wild-type (Fig 4A and 4B). *Pofut1*^*R245A/R245A*^ embryos at E8.5 expressed a little Pofut1 protein (Fig 4A), and there was almost no expression in E8.5 *Pofut1*^*3G/3G*^ embryos (Fig 4A). Pofut1 protein became undetectable in E9.5 *Pofut1*^*R245A/R245A*^ and *Pofut1*^*3G/3G*^ embryos (Fig 4B). *Pofut1* mRNA levels in *Pofut1*^*R245A/+*^, *Pofut1*^*3G/+*^, *Pofut1*^*R245A/R245A*^, *and Pofut1*^*3G/3G*^ embryos were not decreased (Fig 4C), suggesting that the Pofut1 protein level was regulated by post-transcriptional mechanisms. In order to determine if the decreased Pofut1 protein level was caused by an unexpected mutation by the CRISPR/Cas9 system, we amplified the entire coding sequence of *Pofut1* cDNA from total RNA of each embryo, analyzed by direct sequencing, and observed no mutations other than the targeted mutations. The observed phenotype of Notch1 localization may be caused by the decreased protein level rather than the lack of O-fucosylation.

### Pofut1 protein was degraded through a lysosome-dependent protein degradation pathway

**Fig 5:**
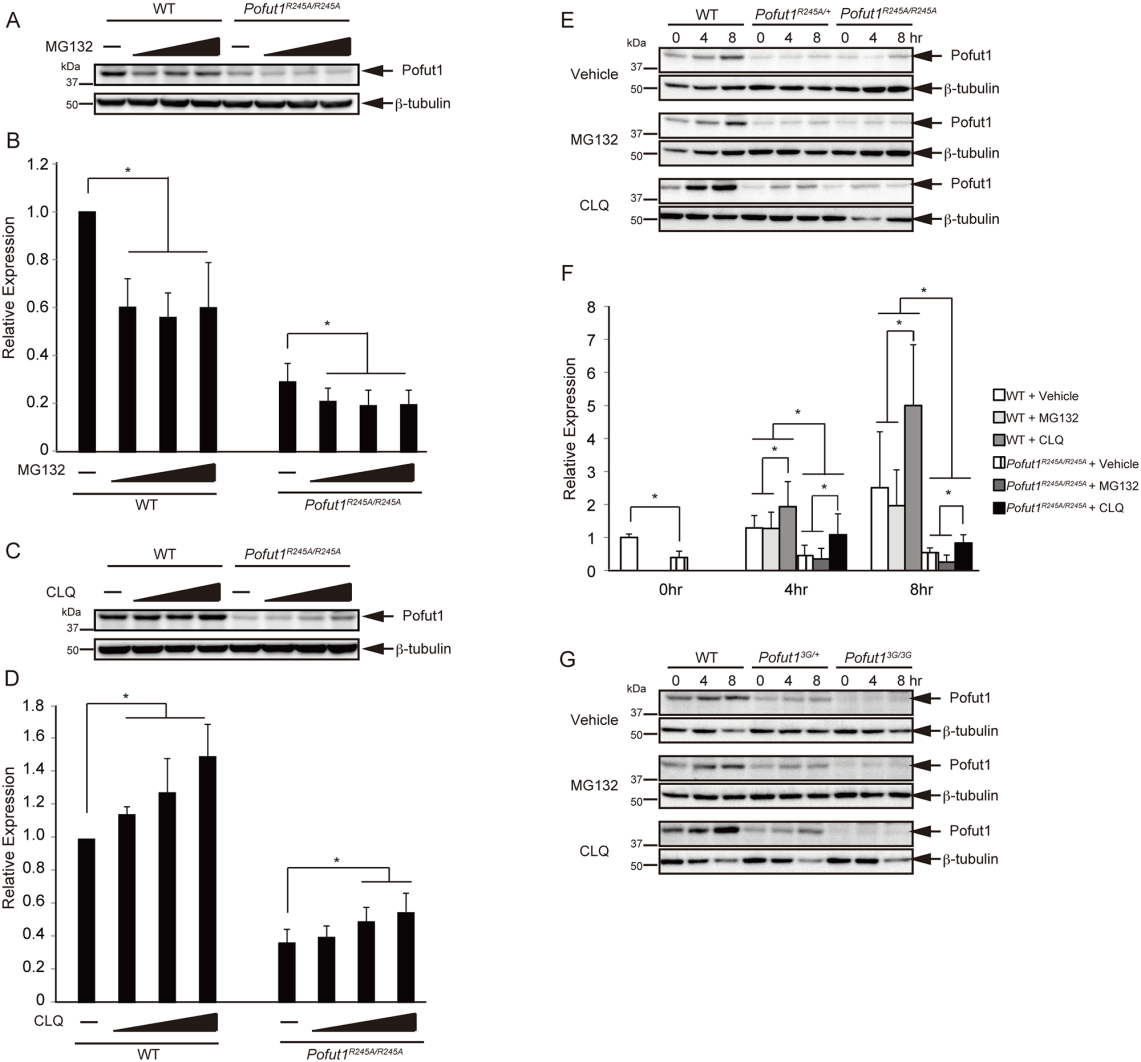
Pofut1 protein was degraded through the lysosome pathway. (A) ES cell lines, which were derived from wild-type and *Pofut1*^*R245A/R245A*^ blastocysts, were treated with a proteasome inhibitor (0, 2.5, 5, or 10 μM MG132) at 8 hr after treatment. These lysates were subjected to western blot analysis with anti-Pofut1 (#28–33) antibody and anti-ß-tubulin antibody. (B) Relative expression of Pofut1 amount in MG132-treated wild-type (n = 6) or *Pofut1*^*R245A/R245A*^ (n = 7) ES cells. Asterisks indicate P<0.05; paired t-test. (C) Wild-type and *Pofut1*^*R245A/R245A*^ ES cell lines were treated with a lysosome inhibitor (0, 1, 2.5, or 5 μM Chloroquine; CLQ), and harvested at 8 hr after treatment. These lysates were subjected to western blot analysis with anti-Pofut1 (#28–33) antibody and anti-ß-tubulin antibody. (D) Relative expression of Pofut1 amount in CLQ-treated wild-type (n = 5) or *Pofut1*^*R245A/R245A*^ (n = 7) ES cells. Asterisks indicate P<0.05; paired t-test. (E) Wild-type, *Pofut1*^*R245A/+*^, and *Pofut1*^*R245A/R245A*^ ES cell lines were treated with a proteasome inhibitor (10 μM MG132) or lysosome inhibitor (5 μM CLQ), and harvested at the indicated time points. These lysates were subjected to western blot analysis with anti-Pofut1 (#35–8) antibody and anti-ß-tubulin antibody. (F) Relative expression of Pofut1 amount in vehicle, MG132, or CLQ-treated wild-type (n = 6) or *Pofut1*^*R245A/R245A*^ (n = 4) ES cells. Asterisks indicate P<0.05; paired t-test. (G) ES cell lines from WT, *Pofut1*^*3G/+*^, and *Pofut1*^*3G/3G*^ blastocysts were treated with MG132 or CLQ, harvested at the indicated time points, and subjected to western blot analysis with anti-Pofut1 (#35–8) antibody and anti-ß-tubulin antibody.

In order to examine why Pofut1^R245A^ and Pofut1^3G^ proteins became unstable, we established ES cell lines from each *Pofut1* mutant, and conducted biochemical analyses. Protein stability is mainly regulated by two mechanisms, the ubiquitin-proteasome pathway or the lysosome-dependent pathway. If the decrease in Pofut1 protein was due to destabilization of the protein, inhibition of either pathway would stabilize the Pofut1 protein. After depleting feeder cells, each ES cell line was seeded and treated with proteasome inhibitor, MG132, or lysosome inhibitor, Chloroquine (CLQ), and then the Pofut1 protein amount in each ES cell line was examined by western blot. Pofut1 protein amounts in both wild-type and *Pofut1*^*R245A/R245A*^ ES cells were decreased when treated with MG132 regardless of the concentration (Fig 5A and 5B). On the other hand, Pofut1 protein amounts in both wild-type and *Pofut1*^*R245A/R245A*^ ES cells were increased upon CLQ treatment in a dose-dependent manner (Fig 5C and 5D). These inhibitor’s inhibitory effects were confirmed by examining ß-catenin (Panel A in S6 Fig) and Notch1 protein amounts (Panel B in S6 Fig), as these proteins were reported to be degraded through the proteasome- and the lysosome-dependent pathway, respectively [44, 45]. Time course experiments showed that the Pofut1 protein amount was gradually increased by an unknown mechanism after seeding to plates (Fig 5E–5G). This increase was also observed in heterozygous cells (Fig 5E and 5G), regardless of treatment with or without DMSO as the vehicle control. MG132 treatment slightly reduced or did not affect Pofut1 protein levels in ES cell lines of any genotype, whereas CLQ treatment increased the Pofut1 protein level in wild-type and heterozygous ES cell lines (Fig 5E and 5G). In *Pofut1*^*R245A/R245A*^ ES cells, the Pofut1 protein level also increased upon CLQ treatment. In contrast, the Pofut1 protein level in *Pofut1*^*3G/3G*^ ES cells was very low, and up-regulation was never observed upon seeding or with CLQ treatment (Fig 5G). As the Pofut1 protein level was partly recovered by CLQ treatment in *Pofut1*^*R245A/R245A*^ ES cells, we tried to examine *O*-fucosyltransferase deficient Pofut1 functions in CLQ treated ES cells by analyzing Notch1 maturation and localization. In WT ES cells, most Notch1 was cleaved at the S-1 position. In contrast, a larger fraction of full-length Notch1 was retained in *Pofut1*^*R245A/R245A*^ ES cells compared with wild-type ES cells (Panels B and C in S6 Fig), as observed in whole embryos (Panel A in S3 Fig). CLQ treatment increased both full-length- and S-1 cleaved-form Notch1 proteins, regardless of the ES cell genotype (Panels B and C in S6 Fig). This data suggests that Notch1 protein was rapidly degraded through a lysosome-dependent pathway independently from *O*-fucosylation. Indeed, Notch1 protein accumulated in and around lysosomes when ES cells were treated with CLQ, regardless of the ES cell genotype (Panel D in S6 Fig). Even though Pofut1^R245A^ protein was recovered upon CLQ treatment, subcellular localization of Notch1 protein also changed. Thus, it is difficult to assess if Pofut1^R245A^ protein functions in controlling the subcellular localization of Notch1.

### Relationship between Notch signaling activation and stabilization of Pofut1 protein

In order to analyze the relationship between the Notch signaling activation and Pofut1 protein destabilization, we first examined the Pofut1 protein amount upon gamma-secretase inhibitor, DAPT, treatment with E9.5 stage wild-type embryos, which inhibits Notch signaling activation. Although Notch1 cleavage for NICD production was completely blocked in the PSM of DAPT-treated embryos (Panel A in S7 Fig), the Pofut1 protein amount only slightly decreased (Panels B and C in S7 Fig). Consistent with this result, in the PSM of *RBPjk* mutant embryos, in which the Notch signaling mediated trans-activation is completely abolished, no significant change in the Pofut1 protein amount was observed (Panels D and E in S7 Fig). As the Notch signaling is activated in only some cells in the PSM and the activation is rapidly changed from posterior to anterior, even if the Pofut1 protein amount was altered by the Notch signaling activity, it may not be observed clearly in the PSM lysate. In order to observe the Pofut1 protein amount when Notch signaling was constitutively activated, we established activated-form Notch1 inducible cell lines using wild-type and *Pofut1*^*R245A/R245A*^ ES cells. The Pofut1 protein amount in wild-type ES cells, but not in the mutant ES cells, was slightly increased by the induction of the active-form of Notch1 protein (NICD) (Panels F and G in S7 Fig), suggesting that the Pofut1 protein amount may be regulated partially through Notch signaling activation. However, it is unlikely that lack of Notch signaling activation is the main cause of Pofut1^R245A^ protein reduction.

## Discussion

### Pofut1 functions for Notch1 protein subcellular localization in the PSM

It has been shown that Pofut1 is indispensable for correct localization of Notch1 protein in the PSM, as Notch1 protein was abnormally accumulated intracellularly in the *Pofut1* deficient PSM [26], similar to the *Ofut1* mutant in *Drosophila* [27, 28]. However, it was still unclear in which organelle Notch1 protein was accumulated and how the localization was regulated. Although Notch1 protein partially co-localized with the ER marker KDEL in the *Pofut1*-deficient PSM, a substantial amount of Notch1 protein was observed intracellularly without co-localization with markers for the ER, Golgi, or endosomes by immunohistochemistry analysis (Panels M-P in S2 Fig and [26]). In this study, we conducted immuno-TEM analysis for detailed analysis of Notch1 protein localization, and found that the majority of Notch1 protein accumulated in the ER in the *Pofut1*-deficient PSM. The differing Notch1 localization with each method may be explained simply by a topological difference, as proteins with the ER retention signal, the KDEL peptide, generally localize in the lumen of ER, while we used an anti-Notch1 C-terminal antibody located outside of the ER to detect Notch1 protein localization. Another possibility is that ER-localized proteins may be distributed unevenly in the ER, and each ER marker labels only a portion of the ER structure in the cells, as reported previously [30]. Notch1 protein accumulation in the ER of *Pofut1*-deficient PSM suggests two possibilities regarding Pofut1 function. One is that Pofut1 has chaperone activity for Notch1 protein, and the other is that *O*-fucose modification of Notch1 by Pofut1 works in quality control of Notch1 protein in the mouse PSM, as reported in *Drosophila* [27, 46]. We aimed to address these possibilities, and established two different point mutation lines, both of which disrupted essential residues for Pofut1 *O*-fucosyltransferase activity. However, unexpectedly, Pofut1 protein stability was markedly reduced in these mutants (Fig 4A and 4B), and caused the same phenotypes as in Pofut1 null mutants. Thus, it was not possible to clarify the importance of Pofut1 *O*-fucosyltransferase activity for Notch signaling in this work.

ER-resident chaperone dysfunction has been reported to induce ER-stress [47], and a swollen ER structure was observed in the cells with ER-stress [48]. In the *Pofut1* mutant PSM, we did not observe any abnormalities in ER structure (Fig 3B and 3C), suggesting that, if Pofut1 works as a chaperone, it may only be for a few specific targets. One of the possible candidate proteins other than Notch1 is the Notch1 ligand Dll1. Delta was reported to be modified with *O*-fucose at the EGF-like repeats in its extracellular domain [49]. Therefore, it is likely that Pofut1 and Dll1 associate together in the ER. Furthermore, Dll1 subcellular localization was altered in the *Pofut1* deficient PSM [26, 50]. However, different from Notch1 localization, an adequate amount of Dll1 was observed on the cell surface of the *Pofut1*-deficient PSM [26, 50]. It is possible that Dll1 mis-localization may have occurred due to the lack of transendocytosis, which generally occurs upon Notch1 and Dll1 interaction, caused by the markedly reduced cell surface amount of Notch1 protein. Further detailed analysis of Dll1 localization in the *Pofut1* deficient PSM may reveal a novel Pofut1 function.

### Regulation of Pofut1 protein stability

Unexpectedly, Pofut1 protein stability was markedly reduced in our mutants (Fig 4A and 4B). As the *Pofut1*^*R245A*^ and *Pofut1*^*3G*^ mRNA levels did not change compared with wild-type (Fig 4C), it is likely that the protein reduction was regulated by post-transcriptional machinery. Even though there is a possibility that these mutations may disrupt Pofut1 protein conformation and decrease the stability, we think it is unlikely, at least for the Pofut1^R245A^ mutant, for the following reasons: First, we made two different types of point mutant mice, and both mutant proteins were similarly destabilized. Second, previous reports demonstrated that over-expression of Pofut1^R245A^ mutant proteins driven by exogenous promoters exhibited similar expression levels to wild-type protein in mammalian cells (Panels E-I in S5 Fig and [22]), *Drosophila* cells [20, 27], and transgenic *Drosophila* [27, 30]. Furthermore, physiological expression of Pofut1^R245A^ in *Drosophila* showed only a slight decrease of the protein [46]. Third, crystal structure analysis of the *C*.*elegan*s Pofut1 homologue, CePofut1, revealed that the 240^th^ Arginine (R240) in CePofut1, which corresponds to the 245^th^ Arginine in mouse Pofut1, is the main residue that captures GDP-fucose directly and catalyzes it; thus, R240 is a key residue for *O*-fucosyltransferase activity [29]. The side chain of R240 is not likely to contribute to the conformation of the CePofut1 protein. On the other hand, the DXD-like motif is on an alpha-helix, which composes part of the donor binding site; therefore, 3G mutants may disrupt Pofut1 protein structure. Indeed, analysis using the Protein 3D modeling software Phyre2 [51] supported these predictions, as Pofut1^R245A^ protein did not affect the 3D structure, but the DXD-motif mutation in Pofut1^3G^ protein changed the structure from an alpha-helix to a beta-sheet. Furthermore, we observed lower levels of Pofut1^3G^ protein when it was overexpressed in mammalian cells (Panels E-I in S5 Fig). We could observe some Pofut1 protein in R245A mutant embryos at E8.5 (Fig 4A), as well as in some conditions in ES cells (Fig 5A), but the protein level in 3G mutants was always very low (Figs 4 and 5). Taken together, destabilization of Pofut1^R245A^ protein may be induced by the mechanisms caused by the mutation in the Pofut1 *O*-fucosyltransferase enzymatic core domain. One of the possible mechanisms is that the mutation altered the affinity of Pofut1 protein for *O*-fucosylation acceptor proteins, and the interaction affected the degradation of Pofut1 protein. The other possibility is that Pofut1 activates some signaling pathway through its *O*-fucosyltransferase activity, and the downstream target in turn regulates Pofut1 protein stabilization. In the latter case, the most likely candidate of signaling is the Notch pathway because it was reported that the *O*-fucosyltration of Notch receptors by Pofut1 is important for Notch protein synthesis and trafficking, for increasing the binding affinity of Notch and its ligands, and for activation of Notch signaling [22–24, 46, 52]. We tried to evaluate this possibility, but inhibition of Notch signaling in the PSM using inhibitors or *RBPjk* mutant mice displayed no clear evidence for the necessity of Notch signaling activation for Pofut1 protein stabilization (Panels B-E in S7 Fig). However, there is still a possibility that cyclic activation of the Notch signaling averaged differential Pofut1 protein amounts in the PSM. We tried to examine the correlation between Pofut1 protein stabilization and Notch signaling activation *in vivo* by immunostaining the PSM with Pofut1 and anti-NICD antibodies, but unfortunately, none of the antibodies against Pofut1 could detect endogenous Pofut1 protein specifically. This was consistent with the western blot results with these antibodies in which several non-specific bands stronger than endogenous proteins were displayed (S5 Fig). Another possibility is that Pofut1 stability is regulated via the signal-sending cells that express Notch ligands. As subcellular localization of Notch1 is abnormal in the null and point mutants (Figs 2 and 3), it may affect the reverse signaling in the signal-sending cells independent of Pofut1 *O*-fucosyltransferase activity. Pofut1 *O*-fucosyltransferase activity may be important for activation of reverse signaling in the signal-sending cells because all Notch ligands contain EGF-like repeats, some of which were reported as Pofut1 targets [50, 53]. However, the reverse signaling that is activated in the signal-sending cells upon Notch receptor and ligand interaction is largely unknown. Furthermore, over 100 proteins that contain EGF-like repeats with the *O*-fucosylation consensus sequence have been identified [54]. This suggests that there are more candidates of *O*-fucosylation acceptor proteins or signaling pathways activated by Pofut1 that may be involved in Pofut1 protein regulation. A future study is needed to identify the factors that are involved in regulating the stability of the Pofut1 protein.

### Importance of O-fucosyltransferase activity of Pofut1 in somitogenesis

It has been reported that *O*-fucosylation of the Notch extracellular domain by Pofut1 is important for quality control of Notch protein and its interaction with ligands [22–24, 46, 52], and these functions are likely important for periodic Notch signaling activation during somitogenesis. Another fucntion of Pofut1 *O*-fucosyltransferase activity may be to regulate synchronous activation of Notch signaling during somitogenesis through Lfng and Dll3 functions. Both Lfng and Dll3 have reported inhibitory functions for Notch signaling activation during somitogenesis, but in different ways. Our recent observation revealed that Lfng plays a crucial role in synchronizing Notch signaling in trans through inhibition of Dll1[13], but it was not clear whether the glycosyltransferase activity of Lfng is important for somitogenesis. Analysis of glycosyltransferase activity-deficient *Lfng* mutant mice with a similar strategy to this study would give insight on the importance of glycosyl-modification of Notch1 and Dll1 during somitogenesis. Dll3 is localized in the Golgi and interacts directly with full-length Notch1, inhibiting the signaling in cis by sending Notch1 to lysosomes before Notch1 processing [55, 56]. A recent study reported that Dll3 was glycosylated by Pofut1, followed by Lfng. The Dll3 mutants, which have disrupted Pofut1 target sites, were unable to rescue the somitogenesis defects in Dll3 null mice [53]. These reports support the importance of Dll3 glycosylation for somitogenesis. Taken together, the glycosylation of Notch1, Dll1, and Dll3 by Pofut1 and Lfng may play a crucial role in precisely controlling the activation of Notch signaling during somitogenesis, but further studies are needed to reveal the underlying mechanisms.

## Supporting information

S1 Fig Characterization of F0 generation of *Pofut1*^*R245A*^ and *Pofut1*^*3G*^ mice. (A-D) F0 generation of *Pofut1*^*R245A*^ mice and corresponding direct sequencing results of each *Pofut1*^*R245A*^ mouse are shown. Note that mice in C and D displayed tail and hair defects. (E-H) Direct sequencing results of each F0 generation of *Pofut1*^*3G*^ mice.
(TIF)

S2 Fig Notch1 accumulates in the ER in the Pofut1 deficient PSM. (A-H) Immunohistochemistry of anterior PSM in E9.5 WT (A-D) and *Pofut1*^Δ*/*Δ^(E-H) embryos using anti-Notch1 (green), anti-Pan-cadherin (red: cell surface) antibodies, and Hoechst33324 (blue: Nuclei). (I-P) Immunohistochemistry of anterior PSM in E9.5 WT (A-D) and *Pofut1*^Δ*/*Δ^ (E-H) embryos using anti-Notch1 (green), anti-KDEL (red: ER) antibodies, and Hoechst33324 (blue: Nuclei). The three right panels are magnified images of insets shown in A, E, I, and M. Scale bars = 10 μm (A), and 5 μm (D).
(TIF)

S3 Fig Pofut1 *O*-fucosyltransferase-deficient embryos phenocopied Pofut1 null mice. (A) Whole embryos of indicated genotype at stage E9.5 were used for western blot analysis. An antibody against the Notch1 C-terminal was used; full-length and S-1 cleaved Notch1 are shown. ß-tubulin was used as a loading control.
(TIF)

S4 Fig TEM analysis of *Pofut1* mutant PSM. (A and B) Lower magnification images of TEM shown in Fig 4A–4C. Corresponding position of Fig 4 images are shown in boxes. Scale bar = 2 μm (A). (C and D) TEM analysis of anterior PSM in E9.5 *Pofut1*^Δ*/+*^ (C) and *Pofut1*^Δ*/*Δ^ (D) embryos. Scale bar = 1 μm (C) Abnormal mitochondria are indicated with arrows.
(TIF)

S5 Fig Characterization of anti-Pofut1 antibodies. (A-H) Upper panels show western blot analysis results using mouse monoclonal antibodies against each Pofut1 peptide or (I) anti-Flag antibody. Clone numbers of each antibody are shown at the top of the panels. (A-D) Whole embryos of indicated genotype at stage E9.5 or (E-I) lysate of 293T cells transfected with indicated expression vectors were used. Arrows indicate Pofut1 bands, arrowheads indicate Flag-tagged Pofut1 bands, and asterisks indicate non-specific bands. Note that Flag-Pofut1 3G protein expression was always lower than the other Pofut1 proteins when ectopically expressed in 293T cells. Lower panels show ß-tubulin amount as a loading control.
(TIF)

S6 Fig Notch1 protein is degraded through a lysosome-dependent pathway regardless of Pofut1 mutation. **(A)** WT and *Pofut1*^*R245A/R245A*^ ES cell lines were treated with proteasome inhibitor (0, 2.5, 5, or 10 μM MG132) and harvested at 8 hr after treatment. (B) WT and *Pofut1*^*R245A/R245A*^ ES cell lines were treated with a lysosome inhibitor (0, 1, 2.5, or 5 μM Chloroquine; CLQ), and harvested at 8 hr after treatment. (C) WT and *Pofut1*^*R245A/R245A*^ ES cell lines were treated with or without a lysosome inhibitor (5 μM CLQ), and harvested at the indicated time points. The lysates were used for western blot analysis. (A) An antibody against ß-catenin was used. (B and C) An antibody against the Notch1 C-terminal was used, and full-length and S-1 cleaved Notch1 are shown. Lower panel shows ß-tubulin amount as a loading control. (D) WT, *Pofut1*^Δ*/*Δ,^ and *Pofut1*^*R245A/R245A*^ ES cell lines were treated with or without a lysosome inhibitor (5 μM Chloroquine; CLQ) and a probe for lysosomes (1 μM Lysotracker Red-DND99), then stained with an antibody against Notch1 (green) and Hoechst33342 (blue). Scale bar = 10 μm.
(TIF)

S7 Fig Activation of Notch signaling partially affected Pofut1 protein destabilization. (A and B) Wild-type embryos were cultured with or without gamma-secretase inhibitor (20 μM DAPT) for 8 hrs. (A) PSMs were stained with anti-cleaved Notch1 (NICD) antibody. The outlines of tissue on the section as well as somites are shown with dashed lines. (B) PSMs were lysed and three or four samples were combined together. These lysates were subjected to western blot analysis with anti-Pofut1 (#35–8) antibody and anti-ß-tubulin antibody. (C) Relative expression of Pofut1 amount in vehicle (n = 4) or DAPT-treated (n = 4) embryos. Averages of each treatment are shown as a bar in the graph. Asterisk indicates P<0.05; paired t-test. (D) The PSMs from the indicated genotypes of *RBPJk* mutant embryos were lysed and three or four samples were combined together. These lysates were subjected to western blot analysis with anti-Pofut1 (#28–33) antibody and anti-ß-tubulin antibody. (E) Relative expression of Pofut1 amount in wild-type (n = 4), *RBPJk* ^Δ/+^ (n = 4), and *RBPJk* ^Δ/Δ^ (n = 4) embryos. Averages of each genotype sample are shown as a bar in the graph. (F) Dox-inducible active-form Notch1-expressing ES cell lines were established using WT and *Pofut1*^*R245A/R245A*^ ES cells. These cells were administered doxycycline and examined 12 hrs after administration. These lysates were subjected to western blot analysis with anti-Notch1 C-terminal antibody, anti-Pofut1 (#28–33) antibody, and anti-ß-tubulin antibody. (G) Relative expression of Pofut1 amount after 12-hr induction of the Notch1 active form in wild-type (n = 6) and *Pofut1*^*R245A/R245A*^ (n = 6) ES cells. Asterisk indicates P<0.05; paired t-test.
(TIF)

S1 Table Potential off targets and the primer sets for the analysis. Mismatches from the on-target sequence are shown in bold. Off target mutations in 3 pups with the Pofut1^3G^ allele were examined by direct sequencing. No mutations were found in loci shown in the table.
(DOCX)
