# Supplementary figures and images for: Pofut1 point-mutations that disrupt O-fucosyltransferase activity destabilize the protein and abolish Notch1 signaling during mouse somitogenesis
Source: PLoS One. 2017 Nov 2;12(11):e0187248. doi: 10.1371/journal.pone.0187248 (PMC5667770; doi:10.1371/journal.pone.0187248)

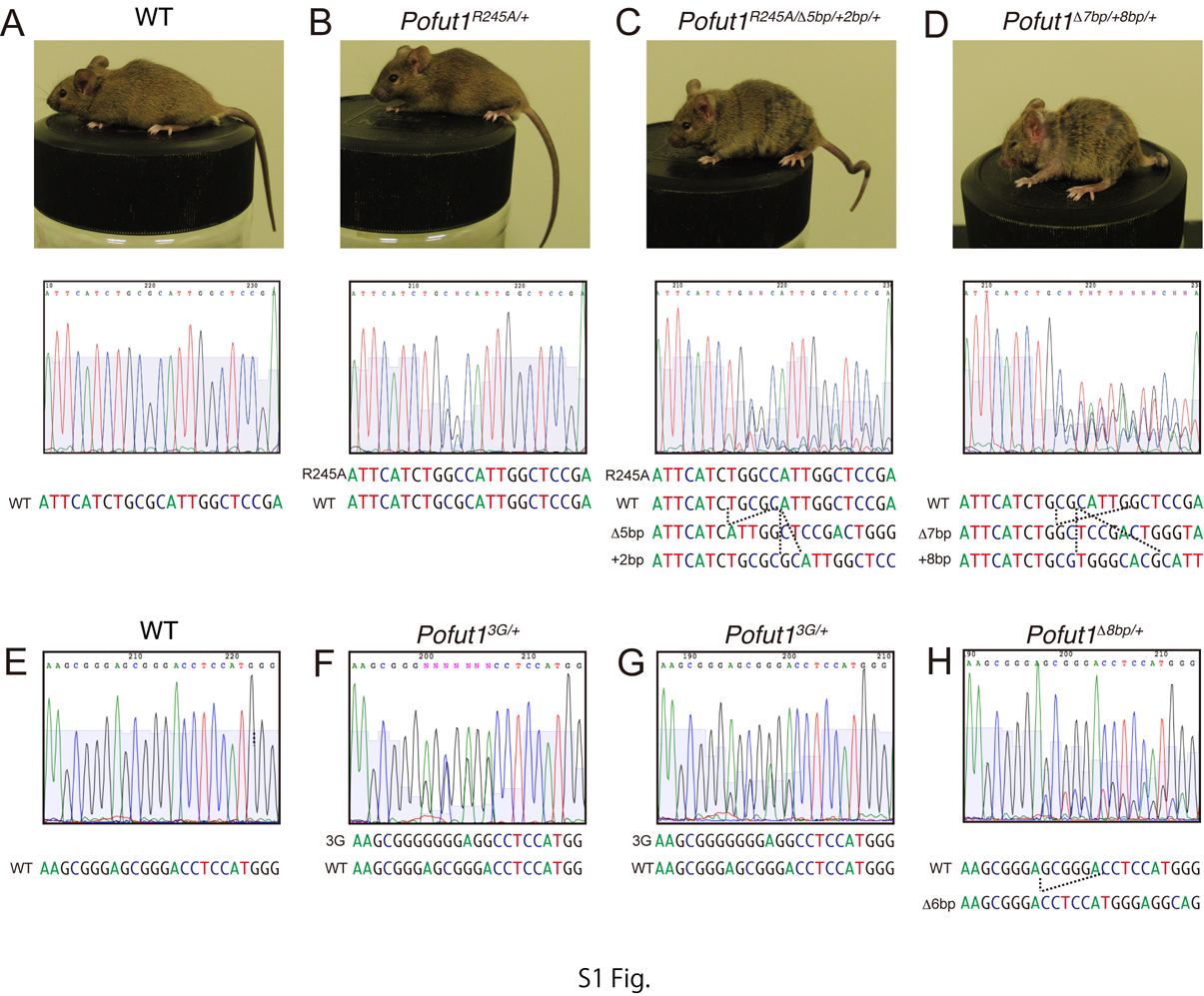

Supplement: S1 Fig — (A-D) F0 generation of Pofut1R245A mice and corresponding direct sequencing results of each Pofut1R245A mouse are shown. Note that mice in C and D displayed tail and hair defects. (E-H) Direct sequencing results of each F0 generation of Pofut13G mice. (TIF) [file pone.0187248.s001.tif]

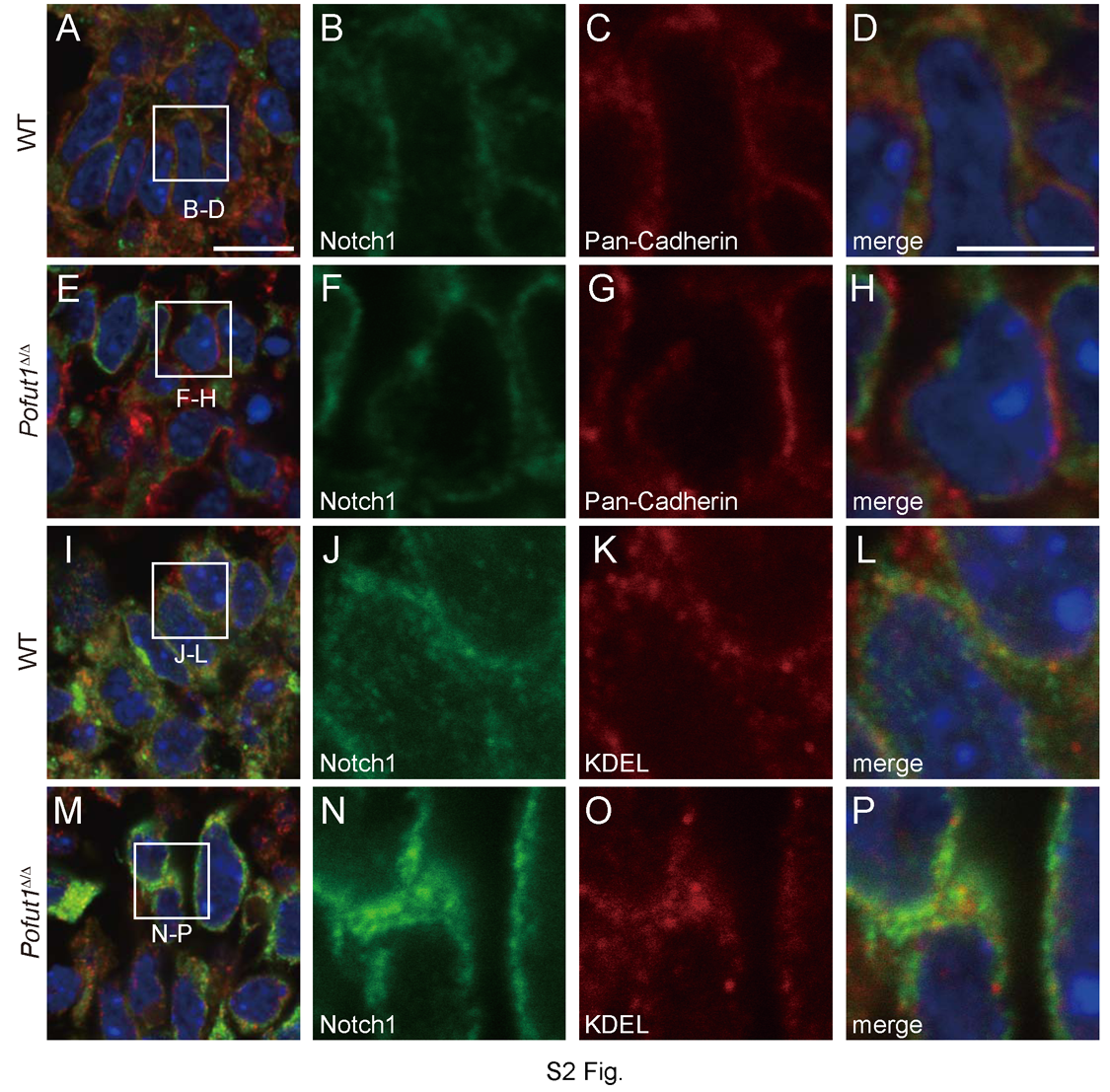

Supplement: S2 Fig — (A-H) Immunohistochemistry of anterior PSM in E9.5 WT (A-D) and Pofut1Δ/Δ(E-H) embryos using anti-Notch1 (green), anti-Pan-cadherin (red: cell surface) antibodies, and Hoechst33324 (blue: Nuclei). (I-P) Immunohistochemistry of anterior PSM in E9.5 WT (A-D) and Pofut1Δ/Δ (E-H) embryos using anti-Notch1 (green), anti-KDEL (red: ER) antibodies, and Hoechst33324 (blue: Nuclei). The three right panels are magnified images of insets shown in A, E, I, and M. Scale bars = 10 μm (A), and 5 μm (D). (TIF) [file pone.0187248.s002.tif]

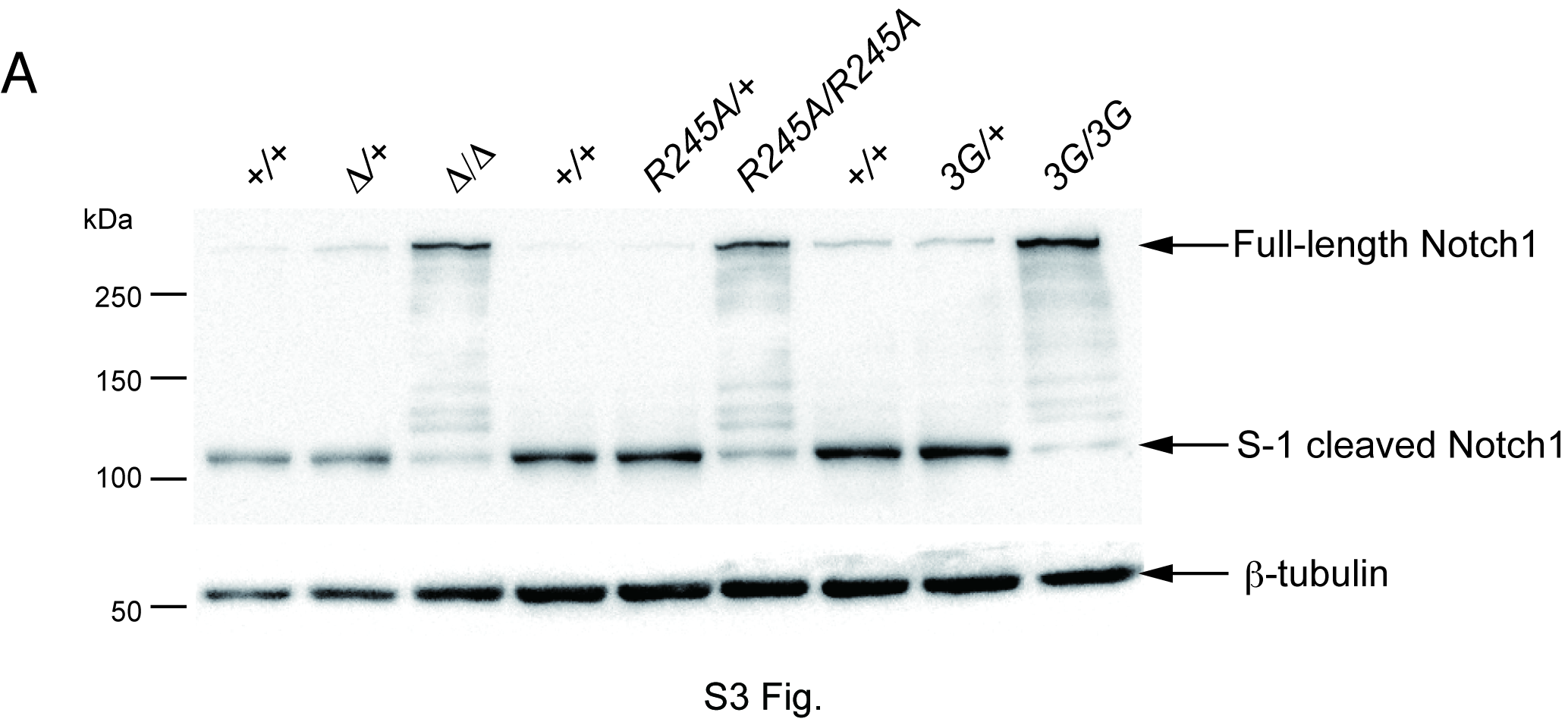

Supplement: S3 Fig — (A) Whole embryos of indicated genotype at stage E9.5 were used for western blot analysis. An antibody against the Notch1 C-terminal was used; full-length and S-1 cleaved Notch1 are shown. ß-tubulin was used as a loading control. (TIF) [file pone.0187248.s003.tif]

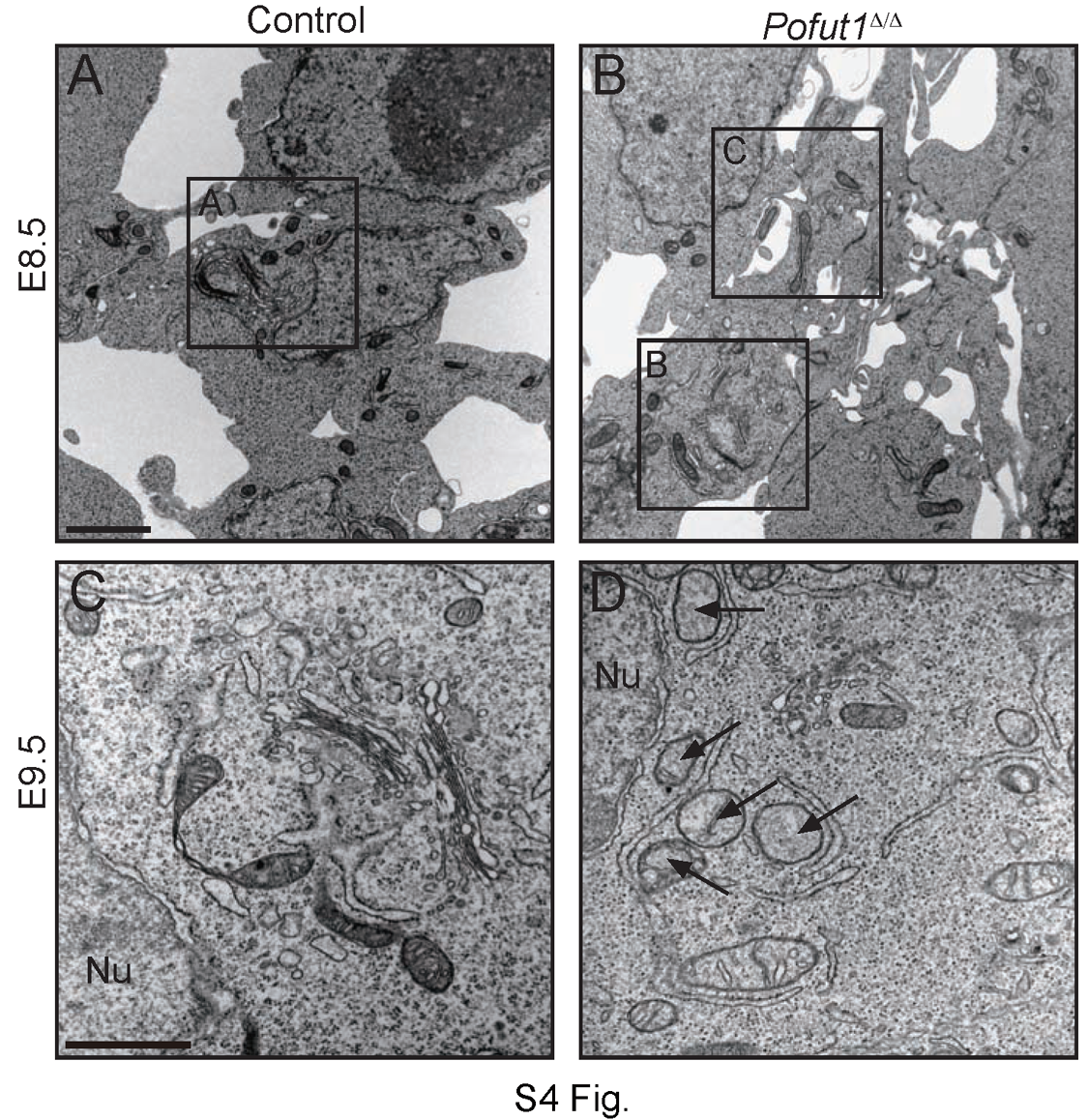

Supplement: S4 Fig — (A and B) Lower magnification images of TEM shown in Fig 4A–4C. Corresponding position of Fig 4 images are shown in boxes. Scale bar = 2 μm (A). (C and D) TEM analysis of anterior PSM in E9.5 Pofut1Δ/+ (C) and Pofut1Δ/Δ (D) embryos. Scale bar = 1 μm (C) Abnormal mitochondria are indicated with arrows. (TIF) [file pone.0187248.s004.tif]

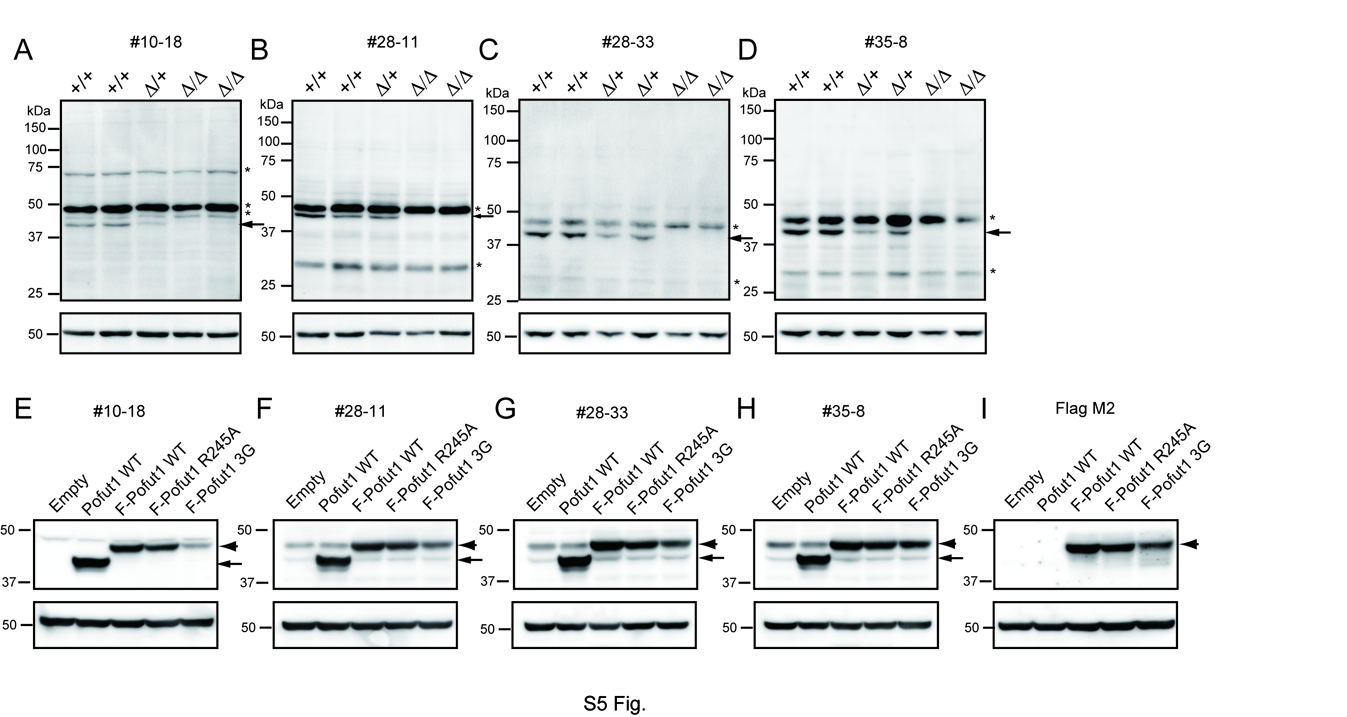

Supplement: S5 Fig — (A-H) Upper panels show western blot analysis results using mouse monoclonal antibodies against each Pofut1 peptide or (I) anti-Flag antibody. Clone numbers of each antibody are shown at the top of the panels. (A-D) Whole embryos of indicated genotype at stage E9.5 or (E-I) lysate of 293T cells transfected with indicated expression vectors were used. Arrows indicate Pofut1 bands, arrowheads indicate Flag-tagged Pofut1 bands, and asterisks indicate non-specific bands. Note that Flag-Pofut1 3G protein expression was always lower than the other Pofut1 proteins when ectopically expressed in 293T cells. Lower panels show ß-tubulin amount as a loading control. (TIF) [file pone.0187248.s005.tif]

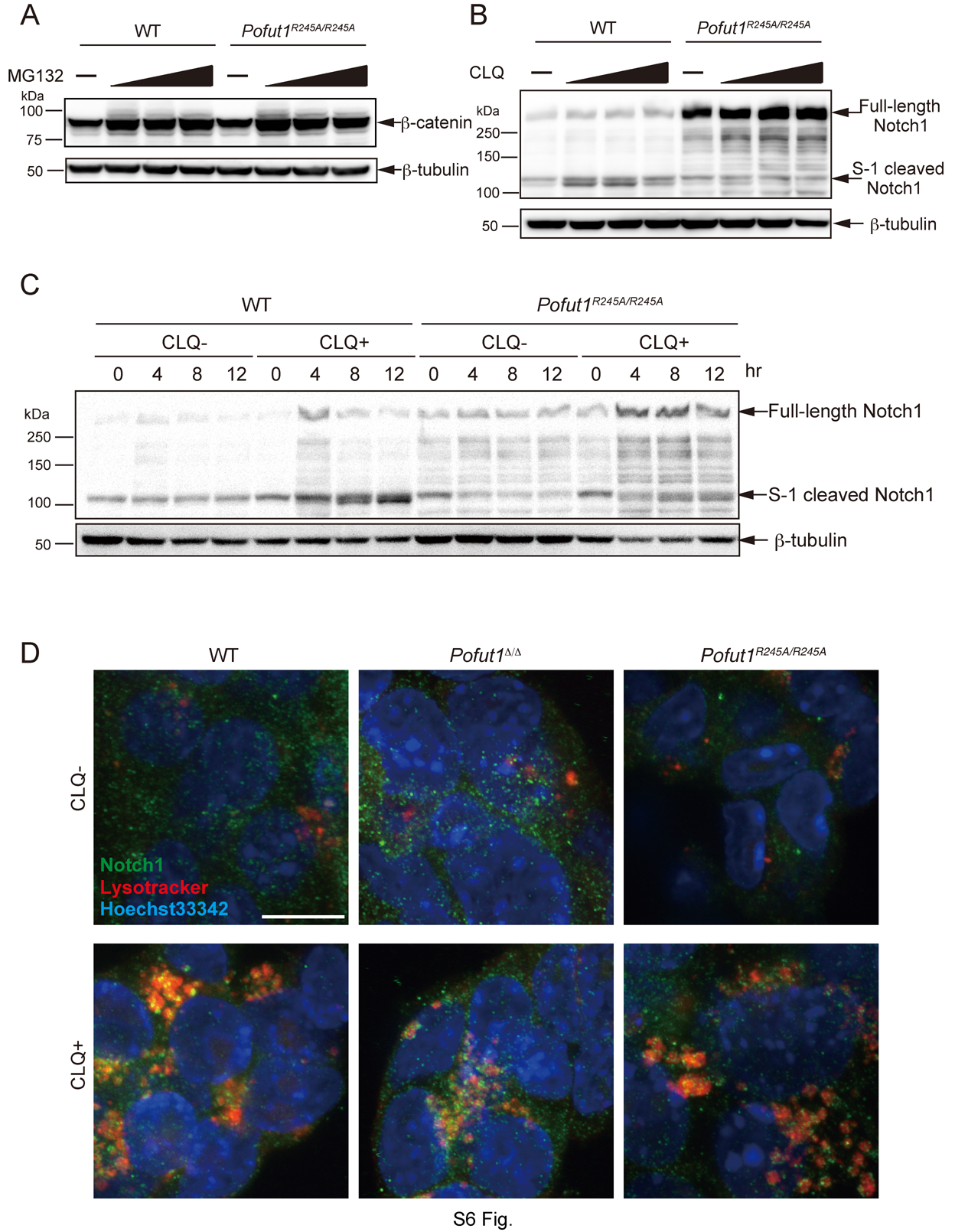

Supplement: S6 Fig — (A) WT and Pofut1R245A/R245A ES cell lines were treated with proteasome inhibitor (0, 2.5, 5, or 10 μM MG132) and harvested at 8 hr after treatment. (B) WT and Pofut1R245A/R245A ES cell lines were treated with a lysosome inhibitor (0, 1, 2.5, or 5 μM Chloroquine; CLQ), and harvested at 8 hr after treatment. (C) WT and Pofut1R245A/R245A ES cell lines were treated with or without a lysosome inhibitor (5 μM CLQ), and harvested at the indicated time points. The lysates were used for western blot analysis. (A) An antibody against ß-catenin was used. (B and C) An antibody against the Notch1 C-terminal was used, and full-length and S-1 cleaved Notch1 are shown. Lower panel shows ß-tubulin amount as a loading control. (D) WT, Pofut1Δ/Δ, and Pofut1R245A/R245A ES cell lines were treated with or without a lysosome inhibitor (5 μM Chloroquine; CLQ) and a probe for lysosomes (1 μM Lysotracker Red-DND99), then stained with an antibody against Notch1 (green) and Hoechst33342 (blue). Scale bar = 10 μm. (TIF) [file pone.0187248.s006.tif]

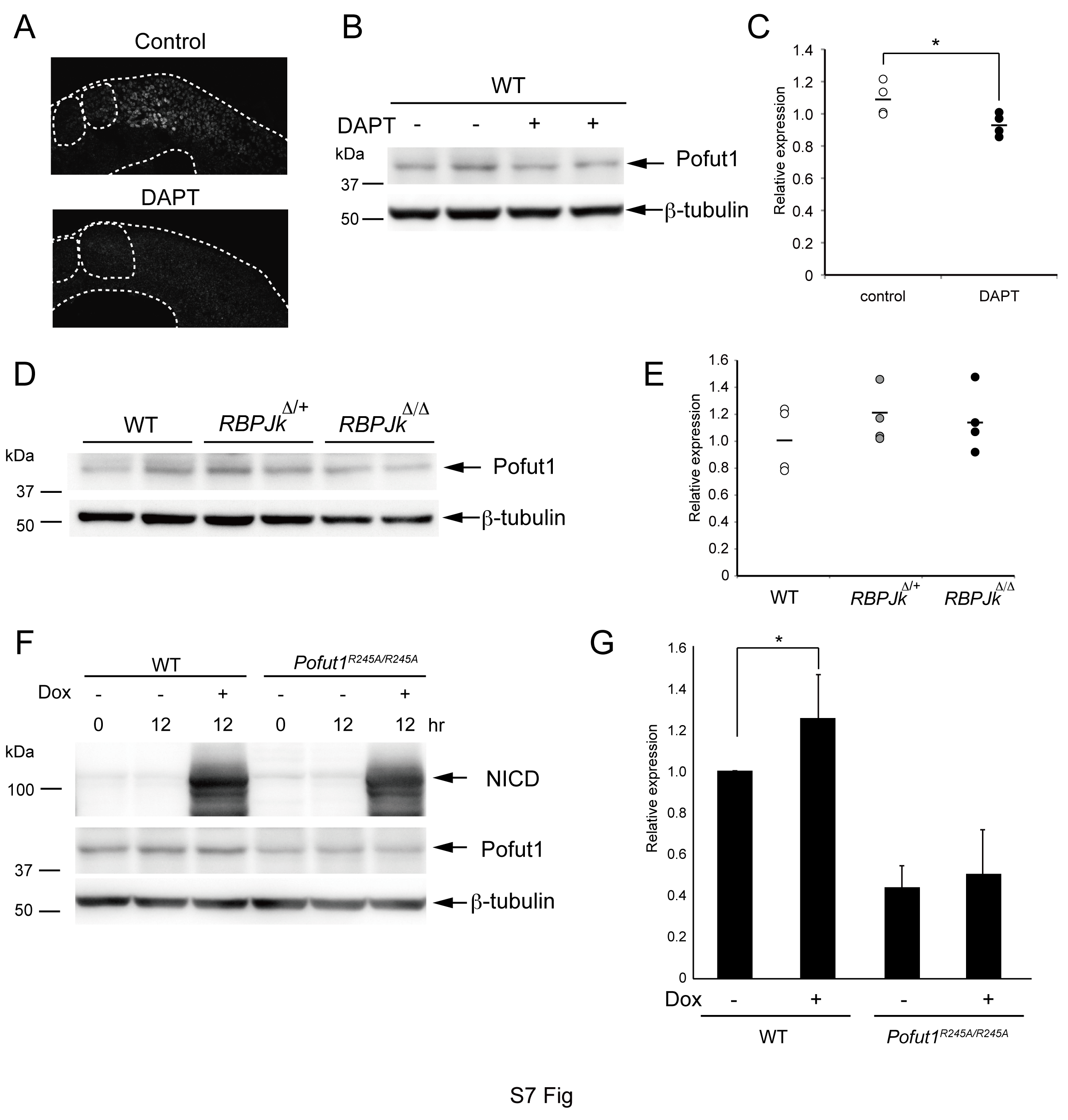

Supplement: S7 Fig — (A and B) Wild-type embryos were cultured with or without gamma-secretase inhibitor (20 μM DAPT) for 8 hrs. (A) PSMs were stained with anti-cleaved Notch1 (NICD) antibody. The outlines of tissue on the section as well as somites are shown with dashed lines. (B) PSMs were lysed and three or four samples were combined together. These lysates were subjected to western blot analysis with anti-Pofut1 (#35–8) antibody and anti-ß-tubulin antibody. (C) Relative expression of Pofut1 amount in vehicle (n = 4) or DAPT-treated (n = 4) embryos. Averages of each treatment are shown as a bar in the graph. Asterisk indicates P<0.05; paired t-test. (D) The PSMs from the indicated genotypes of RBPJk mutant embryos were lysed and three or four samples were combined together. These lysates were subjected to western blot analysis with anti-Pofut1 (#28–33) antibody and anti-ß-tubulin antibody. (E) Relative expression of Pofut1 amount in wild-type (n = 4), RBPJk Δ/+ (n = 4), and RBPJk Δ/Δ (n = 4) embryos. Averages of each genotype sample are shown as a bar in the graph. (F) Dox-inducible active-form Notch1-expressing ES cell lines were established using WT and Pofut1R245A/R245A ES cells. These cells were administered doxycycline and examined 12 hrs after administration. These lysates were subjected to western blot analysis with anti-Notch1 C-terminal antibody, anti-Pofut1 (#28–33) antibody, and anti-ß-tubulin antibody. (G) Relative expression of Pofut1 amount after 12-hr induction of the Notch1 active form in wild-type (n = 6) and Pofut1R245A/R245A (n = 6) ES cells. Asterisk indicates P<0.05; paired t-test. (TIF) [file pone.0187248.s007.tif]
