# Supplementary material for: Pofut1 point-mutations that disrupt O-fucosyltransferase activity destabilize the protein and abolish Notch1 signaling during mouse somitogenesis
Source: PLoS One. 2017 Nov 2;12(11):e0187248. doi: 10.1371/journal.pone.0187248 (PMC5667770; doi:10.1371/journal.pone.0187248)
Supplement: S1 Table — Mismatches from the on-target sequence are shown in bold. Off target mutations in 3 pups with the Pofut13G allele were examined by direct sequencing. No mutations were found in loci shown in the table. (DOCX) [file pone.0187248.s008.docx]

**S1 Table. Potential off targets and the primer sets for the analysis**

| Site name | Primer sequence (5’ to 3’) | Target sequence | UCSC gene | Locus |
| --- | --- | --- | --- | --- |
| Pofut1 3G | ccaatcttcatcctctccaacct | TGCCTTCGTGAAGCGGGAGC GGG | Pofut1 | chr2:+153091528 |
|  | ggctgctttgtatgtgcatgat |  |  |  |
| OT1 | tatgcaccctgttccacagaagt | **ATA**CTTC**C**TGAAGCGGGAGC GGG |  | Chr9:-41008789 |
|  | gaacaccaagccctagctctcat |  |  |  |
| OT2 | cttagcacaaaggctgtcctgtc | TGCC**A**T**T**GTG**G**AGCGGGAGC GAG | smoothened | Chr6:+29703598 |
|  | gactgtgggttacagcttggatg |  |  |  |
| OT3 | ggacgattgggatgaaggtgtat | TGCCTTCGTG**G**AGCGGG**T**GC TGG | Kcnk6 | Chr7:-30017296 |
|  | ctggagctcccggaactaaaag |  |  |  |
| OT4 | cacatcatcctccacttccacat | **G**GCC**AAA**GTGAAGCGGGAGC GGG | Iffo1 | Chr6:+125096007 |
|  | gagccaccagtctgacttgagaa |  |  |  |
| OT5 | cccaggtgcacgttttaattcta | TG**G**CTT**G**GTGAAGC**T**GGAGC CAG | C2cd2 | Chr16:+98080718 |
|  | acagaaaggtctccaccagaaca |  |  |  |
| OT6 | cctggttctgactgctttgctt | **G**GC**G**T**G**CGTGA**G**GCGGGAGC GGG | Drg2 | Chr11:+60268359 |
|  | gggacaagctgttttgtgagatg |  |  |  |
| OT7 | ccccctagttcggactattcattc | TGCC**AGT**GTG**G**AGCGGGAGC CAG | Musashi2 homologue | Chr11:-88580269 |
|  | tgacccctacaagcccactttat |  |  |  |
| OT8 | gctgtcccatgatgaccagtaac | T**C**CCTT**G**G**G**GA**G**GCGGGAGC AAG | Smoc1 | Chr12:+82287324 |
|  | cctaggaagtacacaccccaagg |  |  |  |

Mismatches from the on-target sequence are shown in bold.

Off target mutations in 3 pups with the Pofut1^3G^ allele were examined by direct sequencing. No mutations were found in loci shown in the table.
